# Biomonitoring of pesticides, pharmaceuticals and illicit drugs in a freshwater invertebrate to estimate toxic or effect pressure

**DOI:** 10.1016/j.envint.2019.04.038

**Published:** 2019-08

**Authors:** Thomas H. Miller, Keng Tiong Ng, Samuel T. Bury, Sophie E. Bury, Nicolas R. Bury, Leon P. Barron

**Affiliations:** a Department of Analytical, Environmental & Forensic Sciences, School of Population Health & Environmental Sciences, Faculty of Life Sciences and Medicine, King's College London, 150 Stamford Street, London SE1 9NH, UK; b St Olaves Grammer School, Goddington Lane, Orpington, BR6 9SH, UK; c Department of Pyschology, Royal Holloway, University of London, Egham, Surrey TW20 0EX, UK; d School of Science, Technology and Engineering, University of Suffolk, James Hehir Building, University Avenue, Ipswich, Suffolk IP3 0FS, UK; e Suffolk Sustainability Institute, University of Suffolk, Waterfront Building, Neptune Quay, Ipswich IP4 1QJ, UK

**Keywords:** Exposome, Pesticides, Pharmaceuticals, Environmental risk assessment

## Abstract

Multiple classes of environmental contaminants have been found in aquatic environments, globally. Understanding internalised concentrations in the organism could further improve the risk assessment process. The present study is concerned with the determination of several contaminant classes (107 compounds) in *Gammarus pulex* collected from 15 sites covering 5 river catchments across Suffolk, UK. Quantitative method performance was acceptable for 67 compounds including pharmaceuticals, pesticides, illicit drugs and drugs of abuse. A total of 56 compounds were detectable and ranged from <LOQ to 45.3 ng g^−1^, with cocaine and lidocaine being the most frequently detected compounds present in all biota samples (*n* = 66). For surface water, 50 compounds were detectable and ranged from <LOQ to 382.2 ng L^−1^. Additionally, some pesticides currently not approved for use were detected, including fenuron that reached a maximum of 16.1 ng g^−1^. The internal concentrations of pesticides were used to estimate toxic pressure which showed that for the measured pesticides toxic pressure was low ranging from logTU ≤−7 to ≤−2. This methodology was extended to pharmaceuticals and drugs of abuse in a novel approach that proposed the use of pharmacological data (human therapeutic plasma concentrations) to estimate the likelihood of an effect (or effect pressure) to occur based on the internal exposure of the organism. The quantified effect pressure ranged from logEU ≤−9 to ≤1 with haloperidol showing the largest likelihood for an effect. The approach showed that several pharmaceuticals have the potential to elicit effects but further investigation surrounding thresholds for effects would be required. This new approach presented showed potential to be used to improve risk assessment for pharmaceuticals in the environment.

## Highlights

• A method is presented to quantify pesticides, pharmaceuticals and drugs of abuse.
• Cocaine was the most frequently determined compound found in 100% of samples.
• Several pesticides no longer approved for use in the EU were determined.
• Estimates of toxic pressure for detected pesticides was low.
• Effect pressure of drugs showed good potential to prioritise compounds of concern.

## Graphical abstract

**Figure:**
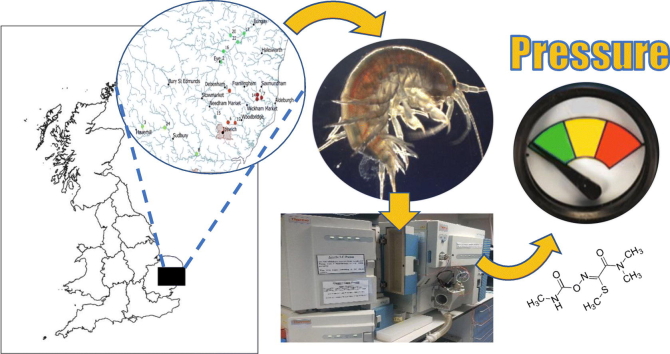

## 1 Introduction

The contamination of the aquatic environment has been the focus of many investigations and many issues have been identified with respect to a number of classes of compounds including pharmaceuticals (Miller et al., 2018) and plant protection products (pesticides) (Barceló, 1991) Within each class, adverse effects of some specific contaminants on biota have been well studied, although effects and/or associated risks are often derived based on exposure concentration levels measured external to the organism (e.g., in water or sediment). A reason for this is that the determination of trace contaminants in biota has traditionally been very challenging, not only in terms of the analytical selectivity required to reliably separate hundreds of different compounds but to do so quantitatively at trace concentrations (e.g. pg-ng g^−1^) (Miller et al., 2018). However, advances in analytical workflows have now enabled trace quantitative measurements in complex biological matrices such that internalised contaminant concentrations can be used to set thresholds for effects (Dodder et al., 2014; Inostroza et al., 2016; Miller et al., 2015).

Arguably, routine determination of internalised concentrations of pharmaceuticals in particular is still critically lacking (Miller et al., 2018). This is also true for some other contaminant classes such as illicit drugs. Additionally, neonicotinoid insecticides, which are largely used on land and have rarely been targeted for measurement in aquatic fauna except for a small number of recent studies in fish and invertebrates (Munz et al., 2018; Jabeen et al., 2015; Masiá et al., 2013). However, other pesticides have been more routinely monitored in aquatic biota, such as organochlorine insecticides, which are reported at the low to mid ng g^−1^ range in both vertebrates and invertebrates (Varol and Sünbül, 2017; Junqué et al., 2018). This is likely due to extensive regulation of these types of contaminants following seminal research in the 1950s (e.g., with dichlorodiphenyltrichloroethane (DDT) (Carson, 2002)) to the more recent Stockholm Convention treaty on persistent organic pollutants which cover many other such compounds (Commission, E., 2004).

Previous studies have used the Species at Risk (SPEAR) index (Beketov et al., 2009; Schäfer et al., 2007) to relate the ‘toxic pressure’ of pesticides in agricultural catchments to the impact on invertebrate communities and is quantified in toxic units (TU) (Liess and Ohe, 2005). The TU is derived from the ratio between the measured concentration of the contaminant in surface water and known toxicity data, such as the LC_50_. Recently, the TU approach has been applied using internal pesticide concentration measurements and predicted internal EC_50_ values (Munz et al., 2018). Aside from pesticides, this approach could also be extended for other contaminant types such as pharmaceuticals. This would prove particularly useful as it would provide an estimate of risk, based on both measured concentrations and effect data. This has already been performed for selected pharmaceuticals in the Antarctic peninsula (González-Alonso et al., 2017). However, a significant barrier to wider application is that there is a paucity of effect data for pharmaceuticals and reported EC_50_ data can vary considerably (de Zwart, 2001). Other approaches such as the use of critical environmental concentrations (CECs) proposed by Fick et al. (Fick et al., 2010), which are based on the fish plasma model (Cook et al., 2003), could be a useful alternative to the use ecotoxicity endpoint data.

The aim of this work was to determine the extent of contaminant occurrence and to estimate the toxic pressure of pesticides and extend this approach to pharmaceuticals, drugs of abuse and illicit drugs to determine an ‘effect pressure’ across several watercourses in Suffolk. This was achieved through the development of an extended analytical methodology to reliably quantify several classes of contaminants in both surface waters and a freshwater invertebrate species (*Gammarus pulex*). Samples were collected from 15 sites covering five river catchments and used to estimate toxic/effect pressure. Internalised concentrations determined herein and a previously developed model for prediction of bioconcentration factors in *G. pulex (*Miller et al., 2019) along with the well-established EPISuite (Agency, U.S.E.P., 2019) BCF predictions in fish were used to calculate internal toxic units (TU_int_) and effect units (EU_int_) for pesticides and pharmaceuticals, respectively.

## 2 Materials and methods

### 2.1 Reagents, chemicals and consumables

HPLC grade methanol, acetonitrile, and LC-MS grade (Optima™) ammonium acetate were purchased from Fischer Scientific (Loughborough, UK). A total of 141 compounds were used in this study (see Supplementary Information (SI)). Of these, 85 were pharmaceuticals/illicits, 22 were pesticides and 34 were stable isotopically labelled internal standards (SIL-IS). All analytical standards were of a purity of ≥97%. Ultra-pure water was obtained from a Millipore Milli-Q water purification system with a specific resistance of 18.2 MΩ cm or greater (Millipore, Bedford, MA, USA). Stock solutions (1 mg mL^−1^) were prepared in methanol or acetonitrile and stored in silanised amber vials (20 mL). Working solutions were prepared daily in ultra-pure water, as required. All solutions were stored at −20 °C and in the dark to reduce possible degradation.

### 2.2 Sample collection

**Fig. 1:**
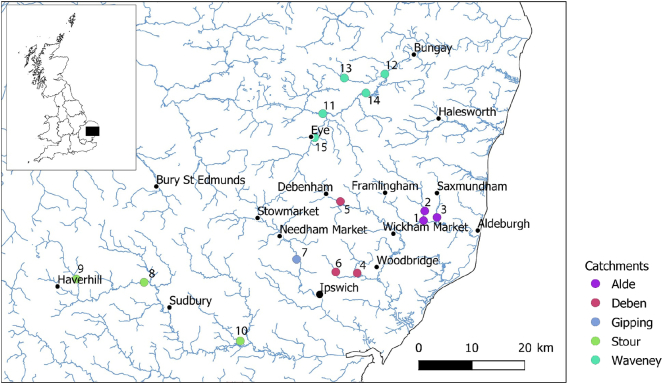
Sampling locations of collected biota and surface water samples within the respective river catchments of Suffolk. Black dots indicate urbanised areas.

Samples were collected in July 2018. Locations were chosen based on previous Environment Agency sampling sites in catchments of the river Alde, Waveney, Stour, Gipping and Deben (Fig. 1). Macroinvertebrates were collected by kick sampling into a 250 μm net. *G. pulex* was present at all sites except the River Box in the Stour catchment and one site on the River Waveney, where the most abundant macroinvertebrate *Ephemera vulgata* (larvae) and *Asellus aquaticus* was sampled instead. At the site on the river Gipping, *G. pulex* numbers were low and the caddis fly *Hydropyshe pellucidula* (larvae) were also sampled. Macroinvertebrates were sorted on site, excess water removed by tissue paper and immediately frozen on dry ice. Samples were kept at −80 °C prior to processing. Water pH and temperature were measured (Table S3) and a 500 mL water sample taken, acidified (0.1% HCl) and stored at 4 °C for a maximum of 4 days prior to analysis to improve stability of analytes as shown in previous studies (Baker and Kasprzyk-Hordern, 2011; Aboulfadl et al., 2010).

### 2.3 Sample preparation

Prior to extraction, frozen *G. pulex* samples were lyophilised at −50 °C under vacuum for 24 h. Pooled samples of 5–6 organisms were placed into 2 mL Eppendorf tubes with a 3 mm diameter tungsten carbide bead and subsequently ground into a fine powder using a TissueLyser LT (Qiagen, Hilden, Germany) set at 50 Hz for 5 min. Freeze-dried composite samples of *G. pulex* material (20 mg) were transferred to a new 2 mL Eppendorf tube with any necessary spiking of standards or SIL-IS carried out directly onto the solid matrix using a 100 μL volume of an appropriate working solution before proceeding with the extraction. A 2 mL volume of 3:1 (MeCN:H_2_O) acidified with 0.1% (*v*/v) glacial acetic acid was added to the material and agitated for 5 min at 50 Hz in the TissueLyser LT. The samples were then placed in an ultrasonic bath for 15 min followed by centrifugation for 5 min at 14,000 rpm to pellet insoluble particulate matter. Following extraction and settling, an aliquot of the supernatant (1.9 mL) was diluted to 100 mL with 10 mM ammonium acetate in ultra-pure water (pH 6.5). Tandem solid phase extraction (SPE) was then carried out on the diluted sample using a Strata Alumina-N cartridge (6 mL, 1 g, Phenomenex Ltd., Cheshire, UK) coupled to an Oasis HLB cartridge (6 mL, 200 mg, Waters Corp., Hertfordshire, UK). Tandem SPE was utilised to remove interfering pigments and lipids (alumina) and pre-concentrate target analytes (HLB). Before loading of the sample, the combined SPE cartridges were first conditioned with 6 mL of methanol and 6 mL of ultra-pure water with 10 mM ammonium acetate. After sample loading, both cartridges were then washed with 1 mL ultra-pure water and dried for ~30 min under vacuum. Cartridges were then stored at −20 °C until analysis. Cartridges were eluted with 5 mL MeOH and dried under pure nitrogen (1.0 bar) at 35 °C using a TurboVap LV (Biotage, Uppsala, Sweden). Extract residues were reconstituted in 0.1 mL 90:10 (*v*/v) 10 mM ammonium acetate in H_2_O:MeCN (optimised). Surface water samples were filtered through a 0.45 μm glass-fibre filter and split into three aliquots (100 mL). Surface water samples then underwent SPE and reconstitution as described above, but without use of the Strata Alumina-N cartridges (as pigments were not problematic). Any necessary spiking or liquid volume measurements were carried out using positive displacement pipettes (Gilson Microman, Villiers-le-Bel, France).

### 2.4 Instrumental analysis and conditions

Briefly, liquid chromatography (LC) was performed on a Vanquish series LC system (ThermoFisher Scientific, Hemel Hempstead, UK) using a Waters SunFire C_18_ column (3.5 μm, 2.1 mm × 150 mm, Waters Corp., Milford, MA, USA) with a KrudKatcher™ Ultra pre-filter (0.1 mm ID, 0.5 μm filter, Phenomenex, Macclesfield, UK) and a Sunfire C_18_ VanGuard Cartridge (3.5 μm, 2.1 mm × 5 mm) at a flow rate of 0.3 mL min^−1^ and an injection volume of 20 μL. Mobile phases were 90:10 (v/v) 10 mM ammonium acetate in H_2_O:MeCN (A) and 20:80 (v/v) 10 mM ammonium acetate in H_2_O:MeCN (B). The gradient elution profile followed a linear ramp of mobile phase B which increased to 10% at 1 min, 35% at 5.6 min, 40% at 7 min, 50% at 8 min and 100% at 11 min and was held for a further 11 min before returning to initial conditions. *Re*-equilibration time was 3 min resulting in an overall run time of 25 min. Detection and quantification was carried out with a TSQ Vantage triple quadrupole mass spectrometer (Thermo Fisher Scientific, Hemel Hempstead, UK) equipped with an atmospheric pressure interface–heated electrospray ionisation (API-HESI-II) source. Mass spectrometry (MS) was performed in selected reaction monitoring (SRM) mode using positive–negative ionisation polarity switching. See the SI for full details of analytical conditions and method performance testing procedures.

### 2.5 Estimation of toxic and effect pressure

**Fig. 2:**
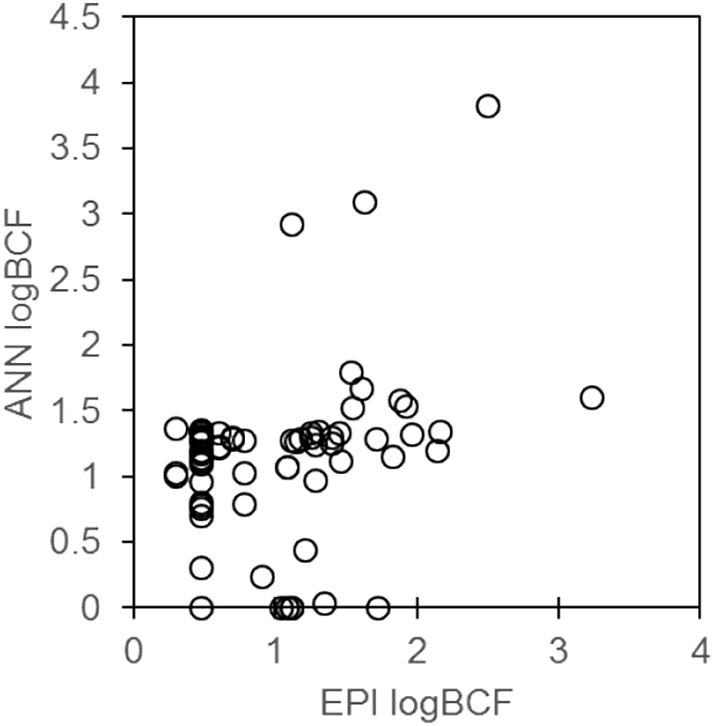
Comparison of predicted logBCF data from EPI suite and ANN model, for individual raw values please see SI Table S5.

Toxic pressure was calculated according to Munz et al. (Munz et al., 2018) using toxic units (TU) to estimate the internal toxic pressure of pesticides. The internal toxic unit (TU_int_) or effect unit (EU_int_) used here is defined by Eqs. (1), (2), (3).

(1) $$\mathrm{EC}50_{\mathrm{int}}=\mathrm{EC}50\times \mathrm{BCF}$$

(2) $${\mathrm{TU}}_{\mathrm{int}}=\frac{C_{i}}{\mathrm{EC}50_{\mathrm{int}}}$$

(3) $${\mathrm{EU}}_{\mathrm{int}}=\frac{C_{i}}{\mathrm{CEC}}$$

where, EC50_int_ is the internal concentration which affects 50% of the population; EC_50_ is the exposure medium concentration affecting 50% of the population; BCF is the bioconcentration factor; *C*_i_ is the concentration of contaminant determined in the organism. For pesticides, available EC_50_ values (48 h acute in *Daphnia magna*) available from the Pesticide Properties Database (Hertfordshire, 2019). The BCFs were estimated from both EPI Suite BCFBAF v3.02 (Agency, U.S.E.P., 2019) software and our own previously developed artificial neural network (ANN) for prediction of BCFs in *G. pulex* (Miller et al., 2019) (Fig. 2). The comparison of the predicted BCFs between both approaches showed relatively good agreement for most cases (see Table S4 and Fig. S1) and overall were not statistically significant (*p-value* = 0.36).

For pharmaceuticals, drugs of abuse and illicit drugs, EC_50_ values were substituted (due to lack of available data) with CECs (Fick et al., 2010). Here, the CEC is the estimated surface water concentration that will give rise to a fish plasma concentration equivalent to the human therapeutic plasma concentration (Eq. 4). Thus, it would be expected and assumed that if drug targets are conserved, an effect would be elicited.

(4) $$\mathrm{CEC}=\frac{H_{T}\mathrm{PC}}{\left(\mathrm{CR}\times P_{\text{blood}:\text{water}}\right)}\;$$

where, H_T_PC is the human therapeutic plasma concentration (μg mL^−1^), CR is the concentration ratio between the human therapeutic plasma concentration and the fish steady-state plasma concentration (assumed to be 1 herein), P_blood:water_ is the partition coefficient of a compound between blood and water.

## 3 Results and discussion

### 3.1 Method performance

Method performance was assessed in *G. pulex* to ensure that the method could reliably quantify targeted analytes at the low ng g^−1^ concentration level (Table 1). A total of 107 compounds were assessed and 67 compounds (55 pharmaceuticals and 12 pesticides) were deemed acceptable for quantification purposes with the remaining analytes suitable for qualitative analysis (according to ICH guidelines). A *t*-test assuming unequal variances showed that there was no significant difference between the performance of the method for either pharmaceuticals or pesticides in terms of recovery and precision (*p* > 0.05). The method showed good sensitivity for trace-analysis with LOQs ranging from 0.09 to 25.2 ng g^−1^ (median: 1.7 ng g^−1^) dry weight and LODs as low as 0.03 ng g^−1^ (median: 0.6 ng g^−1^) dry weight. The sensitivity of the method was comparable to others that have determined pharmaceuticals and pesticides in invertebrates. For example, Inostroza et al., had method quantification limits (MQLs) of 0.01–2.13 ng g^−1^ wet weight (Inostroza et al., 2016), Althakafy et al., reported detection limits ranging 0.04–2.38 ng g^−1^ wet weight (Althakafy et al., 2018) and Munz et al., achieved LOQs of 0.1 to 9 ng g^−1^ wet weight (Munz et al., 2018). Linearity was acceptable (R^2^ > 0.98) and the chromatographic separation showed good reproducibility with an average standard deviation in retention time of ±0.015 min (*n* = 5). The repeatability of the method was also acceptable with average intra-day imprecision of 9 ± 5%, 9 ± 4% and 8 ± 4% at three different concentrations of 25, 50 and 100 ng g^−1^ dry weight. Inter-day precision determined at 50 ng g^−1^ across three days showed slightly lower precision but was still considered acceptable (average 14 ± 4%) and was perhaps due to the inhomogeneity of such small samples and different operators between days. Absolute recoveries of the method ranged from 26 to 100% (average: 74%) and is in line with a recent study that focussed on quantification of both pharmaceuticals and pesticides in *G. pulex* where recovery ranged from 9 to 70% (Munz et al., 2018). Method accuracy averaged 92 ± 10%, 97 ± 12% and 104 ± 9% compared to the expected nominal concentration at 25, 50 and 100 ng g^−1^.

**Table 1:** Method performance assessment for *G. pulex* covering all stages of the analytical workflow. Repeatability was assessed by intra-day (3 concentrations) and inter-day precision (1 concentration) and is expressed by relative standard deviation (RSD). Matrix effects were assessed at 50 ng g^−1^ (*n* = 5) by comparing post-extraction spiked matrix matched standards to a pure analytical standard and negative values indicate suppression effects.

	Matrix Effect						Recovery	Intra-day Precision (%RSD)			Inter-day	Accuracy (%)											LOQ	LOD
	(%)			t_R_ (min)			(%)	25 ng g^-1^	50 ng g^-1^	100 ng g^-1^	Precision (%RSD)	25 ng g^-1^		SD	50 ng g^-1^		SD	100 ng g^-1^		SD	Linearity	Range	ng g^-1^	ng g^-1^
Compound	(n=5)		SD	(n=5)		SD	(n=5)	(n=3)	(n=5)	(n=3)	(n=3)	(n=3)			(n=3)			(n=3)			R^2^	ng g^-1^	(n=6)	(n=6)
4-fluoromethcathinone	-44	±	5	6.92	±	0.014	45	14	13	14	19	75	±	12	92	±	30	117	±	15	0.9930	3.7-500	3.7	1.2
Acetamiprid	-57	±	8	6.92	±	0.001	92	1	15	7	9	102	±	1	98	±	6	96	±	7	0.9997	0.2-500	0.2	0.08
Alprazolam	-46	±	10	10.58	±	0.011	71	8	7	6	13	80	±	7	80	±	12	90	±	6	0.9976	1.2-250	1.2	0.4
Ametryn	-62	±	8	11.76	±	0.002	80	12	8	8	7	72	±	9	111	±	8	96	±	12	0.9950	1.3-250	1.3	0.4
Antipyrin	-52	±	6	5.43	±	0.001	72	1*	10	7	16	84	±	11	125	±	17	126	±	9	0.9973	6.8-500	6.8	1.4
Benzotropine	-70	±	8	11.89	±	0.002	65	9	5	6	15	74	±	7	106	±	12	97	±	5	0.9901	0.6-250	0.6	0.2
Benzoylecgonine	-58	±	2	4.65	±	0.017	71	4	7	8	15	102	±	9	95	±	11	116	±	0	0.9982	0.6-500	0.6	0.2
Betaxolol	-53	±	8	10.29	±	0.014	94	3	11	3	22	100	±	18	88	±	18	80	±	4	0.9944	0.5-250	0.5	0.2
Bezafibrate	-61	±	6	6.96	±	0.016	63	8	13	6	15	89	±	12	97	±	7	108	±	6	0.9984	9.2-500	9.2	3.0
Bisoprolol	-28	±	17	9.54	±	0.066	93	5	6	10	13	85	±	1	101	±	12	86	±	8	0.9898	0.9-250	0.9	0.3
Busipirone	-50	±	9	11.26	±	0.002	76	7	3	6	11	105	±	14	97	±	11	102	±	12	0.9956	1.1-500	1.1	0.4
Carbamazepine	-48	±	8	9.59	±	0.001	67	15	5	8	14	92	±	11	86	±	11	101	±	4	0.9971	0.9-500	0.9	0.3
CBZ_epoxide	-38	±	3	7.70	±	0.001	85	2	12	5	16	92	±	2	113	±	13	89	±	6	0.9988	0.6-500	0.6	0.2
Chloropromazine	-82	±	5	12.66	±	0.008	91	11	5	12	12^a^	99	±	11	98	±	28	140	±	8	0.9928	1.8-500	1.8	5.5
Citalopram	-71	±	5	10.62	±	0.001	76	11	10	13	24	82	±	15	107	±	15	78	±	11	0.9974	2.6-250	2.6	0.9
Cocaine	-37	±	7	9.97	±	0.014	80	8	2	1	9	96	±	7	90	±	11	114	±	12	0.9973	0.5-500	0.5	0.2
Cotinine	30	±	8	3.78	±	0.011	62	15	8	3	13	93	±	21	78	±	19	109	±	3	0.9986	2.6-500	2.6	0.8
Cycluron	-34	±	13	10.73	±	0.002	77	2	5	7	8	109	±	2	100	±	8	104	±	7	0.9997	1.5-500	1.5	0.5
Diazepam	-60	±	10	11.95	±	0.002	87	9	11	8	13	86	±	6	87	±	10	88	±	7	0.9963	0.3-250	0.3	0.1
Dimethmetryn	-50	±	10	13.02	±	0.011	76	5	10	7	7	86	±	3	89	±	2	121	±	8	0.9958	0.1-250	0.1	0.03
Diphenydramine	-51	±	9	10.94	±	0.011	76	12	12	10	16	94	±	11	111	±	16	92	±	8	0.9936	1.8-250	1.8	0.6
Ethirimol	-81	±	4	8.93	±	0.015	81	10	9	11	11	89	±	6	87	±	7	94	±	9	0.9935	1.7-500	1.7	0.6
Fenuron	-56	±	9	6.25	±	0.016	79	9	10	3	11	105	±	12	117	±	10	94	±	3	0.9991	0.6-500	0.6	0.2
Flutamide	-42	±	16	12.27	±	0.002	80	9	7	2	16	90	±	5	96	±	11	75	±	1	0.9942	0.2-250	0.2	0.1
Haloperidol	-79	±	7	11.26	±	0.002	83	2	5	6	16	104	±	2	92	±	17	113	±	8	0.9935	5.3-250	5.3	1.8
Hyrochlorothiazide	-77	±	2	3.90	±	0.011	77	5^a^	13	2	19	100	±	33	85	±	16	118	±	13	0.9952	2.1-500	2.1	0.7
Ketamine	-56	±	6	11.00	±	0.002	54	10	5	7	9	85	±	8	94	±	2	110	±	13	0.9949	1-500	1.0	0.3
Ketoprofen	10	±	11	6.21	±	0.001	69	15	9	10	14	106	±	12	90	±	8	117	±	11	0.9970	15.3-500	15.3	5.0
Ketotifen	-55	±	11	10.74	±	0.002	70	11	8	10	16	81	±	6	104	±	16	99	±	10	0.9955	3.9-250	3.9	1.3
Levamisole	-58	±	4	7.76	±	0.014	61	12	8	6	13	116	±	13	83	±	6	118	±	7	0.9894	4.0-500	4.0	1.3
Levocabastine	-42	±	8	8.16	±	0.015	97	16	10	6	15	71	±	8	97	±	6	113	±	6	0.9934	0.3-250	0.3	0.1
Lidocaine	-46	±	6	11.46	±	0.002	67	1	7	7	13	104	±	1	91	±	8	115	±	8	0.9956	0.7-500	0.7	0.2
Lincomycin	-18	±	5	7.80	±	0.009	82	5	4	9	11	112	±	9	93	±	8	100	±	10	0.9968	4.5-500	4.5	1.5
Lorazepam	-26	±	14	10.44	±	0.013	71	12	10	8	22	81	±	6	85	±	9	118	±	8	0.9895	1.9-250	1.9	0.6
MDMA	-59	±	6	6.10	±	0.042	64	6	9	10	16	111	±	7	101	±	9	97	±	9	0.9995	1.9-500	1.9	0.6
Mephedrone	-12	±	6	7.65	±	0.031	69	12	6	9	14	95	±	8	72	±	13	91	±	3	0.9943	10.5-500	10.5	3.5
Mephosfolan	-49	±	12	11.52	±	0.002	69	7	9	12	7	91	±	14	91	±	3	98	±	11	0.9934	1.4-500	1.4	0.4
Methamphetamine	-61	±	5	6.25	±	0.061	65	4	12	1	12	109	±	16	123	±	17	115	±	10	0.9981	1.7-500	1.7	0.6
Methcathinone	-56	±	1	6.24	±	0.016	43	9	13	2	15	83	±	6	83	±	10	117	±	19	0.9856	3.9-250	3.9	1.3
Methedrone	-63	±	3	6.56	±	0.015	73	1	9	15	11	70	±	1	122	±	8	93	±	15	0.9991	2.9-500	2.9	1.0
Methylphenidate	-74	±	3	9.31	±	0.055	84	15	5	10	11	89	±	11	100	±	15	88	±	8	0.9944	0.2-250	0.2	0.05
Metoprolol	-81	±	12	7.64	±	0.051	84	13	14	5	20	81	±	11	89	±	9	100	±	5	0.9929	2.8-500	2.8	0.9
Nicotine	59	±	17	6.18	±	0.032	51	10	8	13	13	73	±	12	115	±	23	91	±	21	0.9859	2.6-250	2.6	0.9
Nadolol	-20	±	10	5.13	±	0.022	77	5	12	13	12	106	±	18	99	±	2	112	±	10	0.9949	2.2-500	2.2	0.7
Nordiazepam	-13	±	24	11.18	±	0.016	78	7	13	15	15	95	±	5	83	±	9	108	±	5	0.9972	2.4-250	2.4	0.8
Oxamyl	-20	±	23	6.17	±	0.001	90	11	14	18	16	124	±	33	113	±	25	100	±	19	0.9956	1.9-500	1.9	0.6
Oxazepam	-10	±	19	10.19	±	0.012	86	15	12	1	18	81	±	15	122	±	14	76	±	1	0.9948	3.2-250	3.2	1.1
Pirenzipine	-41	±	2	5.33	±	0.016	74	5	3	7	17	82	±	7	97	±	11	119	±	13	0.9917	0.4-500	0.4	0.1
Prometon	-51	±	11	11.22	±	0.002	71	5	2	9	7	92	±	4	104	±	7	100	±	9	0.9971	0.9-500	0.9	0.3
Propamocarb	-65	±	8	5.20	±	0.022	47	3^a^	8	1	8	64	±	5	63	±	5	92	±	13	0.9934	0.6-500	0.6	0.2
Propazine	-43	±	8	11.85	±	0.015	70	7	12	6	13	102	±	9	80	±	3	93	±	19	0.9919	3.5-250	3.5	1.2
Propranolol	-56	±	12	9.96	±	0.015	76	19	15	5	19	74	±	5	74	±	10	107	±	6	0.9990	7.1-250	7.1	2.4
Risperidone	-76	±	4	10.28	±	0.001	73	1	8	5	15	101	±	5	104	±	12	100	±	1	0.9924	0.4-500	0.4	0.1
Rizatriptan	-47	±	5	4.69	±	0.021	55	14	14	8	13	101	±	13	118	±	6	122	±	9	0.9955	3-500	3.0	1.0
Salbutamol	7	±	10	3.30	±	0.016	26	13	10	15	24	88	±	16	96	±	13	117	±	16	0.9987	7-500	7.0	2.0
Sulfadimethoxine	-80	±	3	4.37	±	0.016	76	15	7	6	17	87	±	20	87	±	6	124	±	30	0.9986	0.9-500	0.9	0.3
Sulfamethazine	-75	±	3	4.70	±	0.011	78	6	12	10	23	90	±	13	76	±	15	118	±	12	0.9981	1-500	1.0	2.9
Sulfapyridine	-61	±	7	4.27	±	0.014	77	6	15	12	17	108	±	3	92	±	15	125	±	3	0.9873	3-500	3.0	1.0
Tacrine	-72	±	4	6.51	±	0.060	70	5	12	11	15	93	±	5	104	±	20	85	±	10	0.9912	1.6-500	1.6	0.5
Tamsulosin	-39	±	14	9.91	±	0.001	78	1	15	16	17	72	±	4	121	±	12	84	±	12	0.9976	2.6-500	2.6	0.8
Temazepam	-33	±	12	11.13	±	0.002	81	9	6	9	13	109	±	7	88	±	10	100	±	9	0.9936	0.5-500	0.5	0.2
Thiacloprid	-		-	7.09	±	0.015	100	7	11	3	9	119	±	9	83	±	7	117	±	3	0.9993	0.2-500	0.2	0.07
Timolol	-45	±	3	7.24	±	0.050	72	15	12	10	16	73	±	14	98	±	21	107	±	10	0.9940	25.2-250	25.2	8.3
Tramadol	-67	±	9	8.53	±	0.078	91	10	15	12	16	109	±	12	105	±	14	82	±	10	0.9957	3.1-500	3.1	1.0
Trimethoprim	-67	±	5	6.06	±	0.001	73	20	8	3	16	57	±	20	92	±	11	121	±	15	0.9915	0.1-500	0.1	0.04
Verapamil	-67	±	8	12.03	±	0.002	67	13	8	8	15	82	±	10	109	±	19	117	±	9	0.9904	2.4-250	2.4	0.8
Warfarin	-71	±	4	6.17	±	0.012	75	4	14	9	18	74	±	3	109	±	16	96	±	8	0.9959	0.9-500	0.9	0.3

- not determined.

^a^10 ng g^-1^.

### 3.2 Biomonitoring of emerging contaminants across Suffolk catchments

Occurrence studies are often focussed on the determination of contaminant concentrations in surface water samples and other abiotic matrices such as wastewater and sediment. The limitation of this is approach is that for spot sampling of water, for example, temporal and spatial fluctuations can be considerable and are unlikely to be representative of a chronic exposure scenario. Alternatively, passive sampling that represents a time-weighted average concentration is generally considered semi-quantitative (Mills et al., 2014). Furthermore, these measurements do not accurately represent the real risk to aquatic wildlife as they do not account for bioavailability and it is the internalised xenobiotic concentration that will be the initiating event for any adverse effects. As such, biomonitoring campaigns are now receiving more attention for their importance in determining exposure and hazard (Munz et al., 2018; Shahid et al., 2018).

**Fig. 3:**
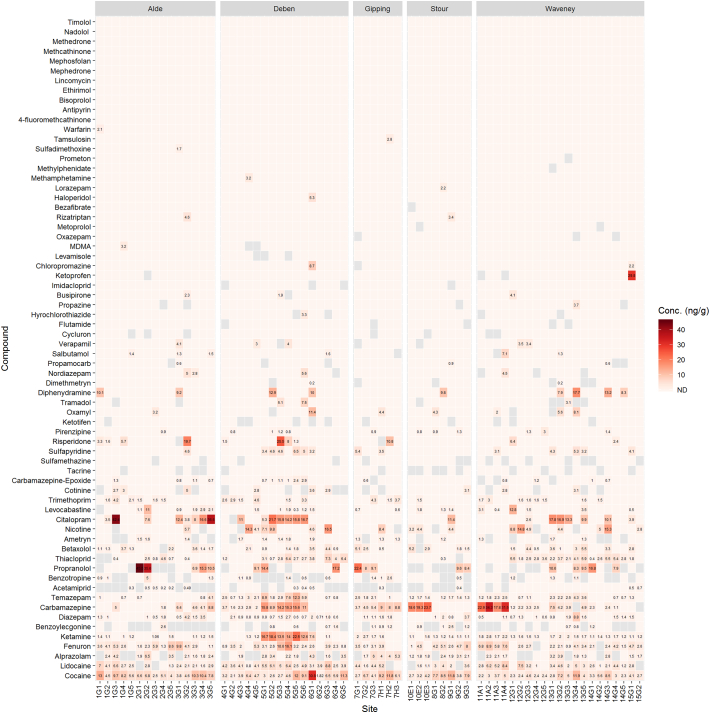
Heatmap of compounds determined in the biological samples that showed acceptable method performance. G, H, A or E indicate the sampled species *G. pulex*, *H. pellucidula*, *A. aquaticus* or *E. vulgata*, respectively. Grey tiles indicate compounds that were detected but below the limits of quantification.

**Fig. 4:**
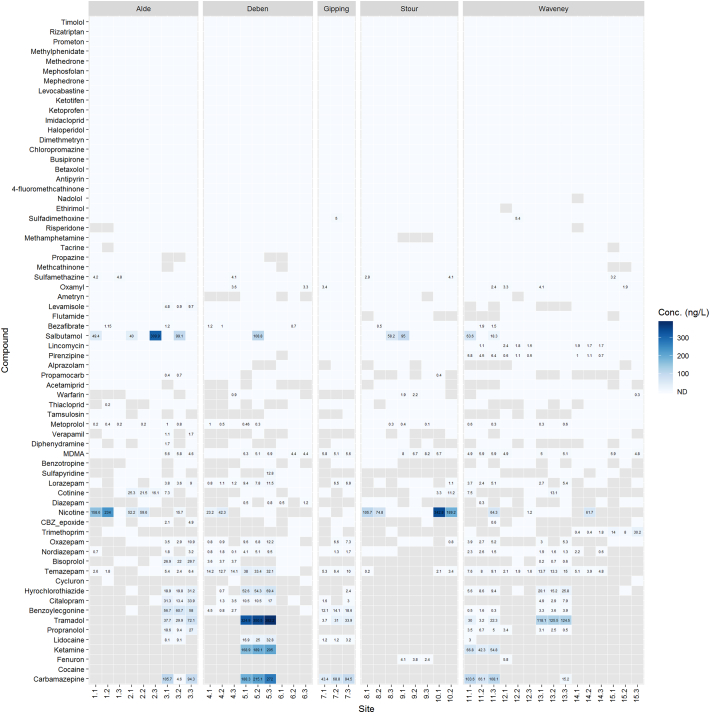
Heatmap of compounds determined in the surface water samples. All sites were samples in triplicate except for Site 10 (*n* = 2). Grey tiles indicate compounds that were detected but below the limits of quantification, decimal points indicate site replicates.

Both water and biota samples were collected across 15 sites in the county of Suffolk. The 15 sites covered 5 different river catchments including Gipping, Alde, Deben, Stour and Waveney. Across the 67 compounds determined, concentrations of compounds were generally very low in both biota samples (parts per billion range) and water samples (parts per trillion range). For biota samples (*n* = 66), the average concentration determined was 4.3 ± 5.2 ng g^−1^, with maximum and minimum concentrations of 45.5 ng g^−1^ (propranolol) and 0.2 ng g^−1^ (acetamiprid), respectively (Fig. 3). In comparison to surface water samples, concentrations averaged 23.8 ± 54.9 ng L^−1^, with the maximum and minimum concentrations of 382.2 ng L^−1^ (tramadol) and 0.1 ng L^−1^ (nordiazepam), respectively (Fig. 4). In general, Site 1 in the Deben catchment showed increased concentrations of compounds such as ketamine, carbamazepine and citalopram compared to the other sites within the same catchment and between the remaining catchments. These higher concentrations also coincide with higher concentrations in surface water for compounds such as ketamine, carbamazepine and tramadol, the source of which is unclear but for these compounds their removal at WWTPs is low (Munro et al., 2019). Debenham is a large village of 2200 inhabitants (Fig. 1) served by a small WWTP upstream of the sample site. The sources for these contaminants are likely to be related to public consumption and output through WWTP effluents (for pharmaceuticals, drugs of abuse and illicits). A previous study that has quantified related compounds in influent and effluent samples from a WWTP in London showed that the concentrations in the surface water determined here are in the range of those determined in effluent (~10–50 ng L^−1^) (Munro et al., 2019). Additionally, spread of sludge and bio-solids including (Barron et al., 2008) reclaimed wastewater for irrigation from WWTPs onto agricultural land could lead to further surface run-off or leaching of pharmaceuticals and controlled substances into surface waters (Carter et al., 2019). For pesticides, run-off and leaching (including possible re-mobilisation) are the potential sources relating the compounds detected herein (Huber et al., 2000).

#### 3.2.1 Illicit drugs, drugs of abuse and life-style related compounds

Interestingly, the most frequently detected and highest concentration compounds in biological samples were illicit drugs and/or drugs of abuse, such as cocaine, ketamine, alprazolam and diazepam. Cocaine was detected and quantified in all biota samples across all 15 sites at an average of 5.9 ± 4.3 ng g^−1^ (max. 30.8 ng g^−1^). Average concentrations of cocaine between different catchments did not vary significantly showing widespread contamination (Alde = 6.9, Deben = 6.9, Gipping = 6.8, Stour = 6.2 & Waveney = 4.2 ng g^−1^). Lidocaine was the second most frequently detected compound in the biota samples that can be used as an adulterant to ‘cut’ cocaine due to its synergistic effects (Barat and Abdel-Rahman, 1996) or is used as local anaesthetic. Another commonly used adulterant for cocaine use is levamisole. This compound, however, was not frequently detected in either biota or surface water samples. However, illicit compounds are rarely monitored in aquatic fauna, with only one previous occurrence study in the literature that determined cocaine at an average concentration of 0.28 ng g^−1^ dw in *Mytilus* spp. (Dodder et al., 2014). A separate investigation into the bioaccumulation potential of cocaine in European eels (*Anguilla anguilla*) in Italy revealed tissue concentrations ranging from 0.47 to 30.5 pg g^−1^ ww depending on tissue type at an exposure concentration of 20 ng L^−1^ (Capaldo et al., 2012). However, eels were not studied as part of this or previous works in our laboratory. The source of the widespread cocaine contamination is unclear. Scattered throughout the catchments of these Suffolk rivers are small wastewater treatment plants that will discharge into the water courses. However, secondary wastewater treatment with activated sludge are efficient at removing cocaine (~90% (Yadav et al., 2017)), whereas trickling filters are less efficient (35–37% removal (Yadav et al., 2017)). The dispersal of deactivated sewage sludge onto farmland as a fertiliser is unlikely to be a primary source and concentrations of cocaine in sludge have been reported as low, at ~3 ng g^−1^ (Petrie et al., 2016). The primary metabolite of cocaine, benzoylecgonine (BZE) was also frequently detected, but often below the LOQ in both water and biological extracts. The concentration of cocaine determined in surface water samples was also below the LOQ for all sites and previous studies in the UK have often determined cocaine at ~1–10 ng L^−1^ in surface water (Munro et al., 2019; Kasprzyk-Hordern et al., 2008). The ratio between cocaine to BZE is also important to consider and may potentially indicate the source of input into the environment. For example, in wastewater analysed from London in 2014, the ratio between cocaine and BZE was 0.51 ± 0.09 in influent, but was very different and more variable in effluents measured on the same days (2.60 ± 1.46) (Munro et al., 2019). Therefore, it is expected that the ratio between cocaine and BZE in river water catchments should be similar to effluent ratios but this was not the case for London, where the ratio for cocaine:BZE over six weeks of daily monitoring was 0.21 ± 0.1 (similar to influent ratios) (Munro et al., 2019). Thus, it is proposed that the input of cocaine into surface waters in the UK is likely due to combined sewer overflow events or leakage from sewer misconnections and cesspit overflow. Interestingly, the ratio in the biota samples measured here (mean: 5.00) indicated that cocaine had preferential accumulation over its demethylated metabolite, BZE.

Tramadol was frequently detected in surface water and reached the highest measured concentration across the sites of 382.2 ng L^−1^. This compound has previously been detected in UK rivers ranging from <30 ng L^−1^ to 5970 ng L^−1^ (Munro et al., 2019; Kasprzyk-Hordern et al., 2008). Effect assessments studies demonstrate lowest observed effects concentrations (LOEC) of 10 μg L^−1^ in fish embryo tests (Sehonova et al., 2017). Occurrence of this compound here was infrequent with a maximum measured concentration of 7.5 ng g^−1^. Field-derived bioaccumulation studies have suggested that bioaccumulation is low with BAFs <5 and tissue concentrations in fish were < 6 ng g^−1^ (Grabicova et al., 2014). Ketamine was also frequently detected in biological and surface water samples here, with concentrations reaching up to 22.5 ng g^−1^ and 205 ng L^−1^. However, to the authors' knowledge, ketamine has not been previously reported in aquatic fauna, but surface water concentrations have been measured at 12 ng L^−1^ (Munro et al., 2019).

The benzodiazepines are a class of compounds used for medicinal purposes but are also misused/abused. Alprazolam, diazepam and temazepam was determined at 2.7 ± 1.3 ng g^−1^, 1.5 ± 1.4 ng g^−1^ and 2.4 ± 2.3 ng g^−1^, respectively. Lorazepam, oxazepam and nordiazepam were infrequently detected. Our previous work has shown that diazepam and temazepam have a low potential to accumulate in *G. pulex* and which are capable of rapid biotransformation and elimination of these compounds (Miller et al., 2017). In surface water samples, diazepam was infrequently detected and often occurred at <1 ng L^−1^. Alprazolam was also infrequently detected and below the LOQ. The average concentrations of the remaining benzodiazepines were 9.0 ± 9.4 ng L^−1^ (temazepam), 5.2 ± 3.5 ng L^−1^ (oxazepam), 4.8 ± 3.3 ng L^−1^ (lorazepam) and 2.2 ± 0.8 ng L^−1^ (nordiazepam).

Synthetic cathinones including methedrone, mephedrone, methcathinone and 4-fluoromethcathinone were not detected at any site in the biota samples. However, methcathinone was detected below the LOQ at a small number of sites in surface water samples from the river catchments of Waveney, Deben and Alde. Cathinones are psychoactive substances and their consumption across the UK and Europe formed the basis of several occurrence studies in surface water and wastewater (Baz-Lomba et al., 2016). Nicotine was determined in surface water samples up to 342.8 ng L^−1^ and was also detected in 38% of the biota samples ranging from <LOQ to 16.5 ng g^−1^. Its primary metabolite, cotinine, was also detected in biota and surface water samples, but less frequently and at lower concentrations. Based on human metabolism, the expected ratio of nicotine to cotinine would range between 0.65 and 1.00 (Benowitz et al., 2009). However, for surface water samples the average ratio of nicotine:cotinine was 7.61 and in biota samples was 2.39. The higher concentration of nicotine to cotinine has been reported previously for effluent wastewater (Bueno et al., 2012) and a similar ratio to surface water can be estimated (6.3) from reported concentrations in influent wastewater samples (Huerta-Fontela et al., 2008). These types of compounds are useful to monitor in the environment as they can serve as indicators of population health and lifestyle choices. Previous studies have identified markers of alcohol consumption such as ethyl sulfate (Ryu et al., 2016). Whilst other sewage epidemiology studies have used drug concentrations in wastewater to relate back to recreational drug use of the population (van Nuijs et al., 2011). In addition to the association with human health, these drugs are often not monitored in biota and so any potential risk from exposed aquatic wildlife is poorly understood. The reason for poor exposure and hazard assessment is likely to stem from that many of these substances are also medicines and therefore will be considered ‘legacy’ products, which do not require ERA. Interestingly, seven of the top ten most frequently detected compounds in biota samples are related to illicit drugs/drugs of abuse. The risk of these compounds is not well understood due to the lack of literature, but as these compounds are all psychoactive, any effects on fauna may be elicited through behavioural changes (Bossus et al., 2014; Brodin et al., 2013).

#### 3.2.2 Pharmaceuticals

The most frequently detected pharmaceutical in both biota and surface water samples was carbamazepine. This compound has been shown to occur in *G. pulex*, surface water and sludges samples (Miller et al., 2015; Barron et al., 2008). Measured concentrations in the biota samples ranged from <LOQ to 31.5 ng g^−1^ and in surface water, the concentrations ranged from <LOQ to 272 ng L^−1^. The highest surface water concentrations were measured at Site 1 (average: 225 ng L^−1^) which also corresponded to relatively high concentrations measured in *G. pulex* with an average of 16.3 ng g^−1^. Higher concentrations of carbamazepine were determined at site 6 and 8 for the *Ephemera vulgata* and *Asellus aquaticus* samples. Site 8 surface water concentration of carbamazepine were below the LOQ and site 16 averaged 92.6 ng L^−1^. This may suggest that *E. vulgata* and *A. aquaticus* are more sensitive than *G. pulex* to the accumulation of carbamazepine. However, surface water concentrations often do not translate well into internal concentrations for several reasons such as temporal variation, spatial variation and migration behaviour of aquatic fauna among other influences. Additionally, the main human metabolite of carbamazepine, CBZ-epoxide, was detected across 30% of the biota samples. This metabolite has been detected and measured in invertebrate species including *G. pulex* and *Mytilus galloprovincialis* showing conservation of biotransformation pathways (Miller et al., 2017; Boillot et al., 2015). The increased concentration of carbamazepine at Site 1 *G. pulex* samples also coincided with increased detection of the epoxide metabolite. However, the metabolite was not detected in *E. vulgata* larvae and was minimal in *A. aquaticus* despite higher concentrations of carbamazepine measured in these species. This may indicate a different sensitivity of these organisms to carbamazepine through toxicokinetics, where biotransformation and elimination routes are different. The mean ratio of carbamazepine to the epoxide metabolite was 8.9 in the biota samples, which is closer to observed human therapeutic ratios of ~5 (Potter and Donnelly, 1998).

The highest measured pharmaceutical concentration across the biota samples alone was for the beta-blocker propranolol (45.5 ng g^−1^ at Site 4). The concentrations of propranolol in surface water ranged from <LOQ to a maximum of 27 ng L^−1^, which is significantly below (two orders of magnitude) the reported no-observed effects (NOEC) and lowest-observed effects (LOEC) in fish and invertebrates (Huggett et al., 2002; Owen et al., 2009). Other beta-blockers were detected at lower concentrations and less frequently which included betaxolol, salbutamol and metoprolol. The remaining beta-blockers included in this method, were not detected at any site for the biota samples (timolol, nadolol and bisoprolol). However, for surface water samples, bisoprolol was detected frequently across all river catchments, with metoprolol and the beta-agonist salbutamol less frequently detected.

The selective serotonin reuptake inhibitor citalopram was frequently detected in biota samples at Site 7, Site 1 and Site 20, with concentrations ranging from 3.8 to 36.6 ng g^−1^. The maximum concentration was determined to be 42.4 ng g^−1^ at Site 14. Surface water concentrations of citalopram were often below the LOQ but were determined at higher average concentrations of 14.7 ± 10.6 ng L^−1^ for Site 1, Site 7 and Site 20. Citalopram has been previously determined up to concentrations of 20.6 ng g^−1^ in bivalves (*Mytilus spp.*) (Álvarez-Muñoz et al., 2015), 0.212 ng g^−1^ in fish brain tissue (*Catostomus commersonii*) (Schultz et al., 2010) and more recently was reported to reach concentrations of ~6000 ng g^−1^ in *Hydropsyche spp* (Richmond et al., 2018). From the literature, citalopram has been observed to have low accumulation factors ranging from less <7 to 47 (Grabicova et al., 2014; Lajeunesse et al., 2011). Based on occurrence data presented here, it would also likely have a low bioaccumulation factor. Furthermore, the analytical method here could not distinguish between the enantiomeric forms of citalopram with the S-enantiomer responsible for the pharmacological action where it has also been suggested that R-enantiomer inhibits this therapeutic effect. Other researchers have shown that racemic mixtures of pharmaceuticals can often be enriched by either human or microbial biotransformation or may remain as racemates if biodegradation does not occur (Evans et al., 2017). Many of the pharmaceuticals reported here display stereoisomerism, which is poorly understood in terms of environmental risk, and is often overlooked in both fate and effect-based studies (Evans et al., 2017). The most frequently detected antibiotic was trimethoprim with measured concentrations ranging from 1.5 to 4.6 ng g^−1^. Other antibiotics detected included three sulphonamides: sulfamethazine; sulfapyridine; and sulfadimethoxine. However, sulfamethazine was not quantifiable in any sample and sulfadimethoxine was only measured once reaching 1.7 ng g^−1^. Bioconcentration studies for sulfamethazine in *Oryzias melastigma* have ranged from <1–145 depending on tissue and biological sex indicating that there is no or little potential for bioaccumulation (Miller et al., 2017; Hou et al., 2003). The low bioaccumulation is likely to stem from the polarity (logP = 0.44, logD_8_ = 0.1) and ionisation state of the drug which has been shown to influence uptake in fish and invertebrates (Miller et al., 2019; Chang et al., 2018; Meredith-Williams et al., 2012). Sulfapyridine, was also infrequently detected except at Site 1, with an average concentration of 4.8 ng g^−1^. The low occurrence of the sulphonamides in biota is likely due to the high polarity and metabolism of these compounds.

#### 3.2.3 Pesticides

Neonicotinoids have gained much attention recently, with the EU now enforcing a near total ban on their use (Commission, E, 2011). Few studies have determined the presence of these compounds in aquatic fauna (Munz et al., 2018; Shahid et al., 2018). Other studies have targeted these pesticides in fish, but ultimately were not detected (Jabeen et al., 2015; Masiá et al., 2013). However, these compounds do occur in surface water and averaged at 130 ng L^−1^ across 19 studies (Morrissey et al., 2015). The compounds thiacloprid and acetamiprid were infrequently detected in surface water samples across all sites here and remained below the LOQ. Imidacloprid was not detected at any site. This agreed with a recent report on neonicotinoid contamination in UK surface waters (Shardlow, 2017), which summarised that thiacloprid and acetamiprid showed low contamination which is likely related to their low use as opposed to other neonicotinoids such as clothiandin and thiamethoxam. The qualitative data showed thiamethoxam was not detected across any sites and clothiandin was infrequently detected. This contrasts data reported for thiamethoxam in the river Waveney which showed concentrations reaching up to 1.03 μg L^−1^ and an average concentration of ~60 ng L^−1^. A possible reason for the disparity between the data reported here is that the previous report was from a monitoring campaign in 2016. The samples collected in the present study were from July 2018, following the driest period record with no rain in the previous 55 days (Office, M, 2018) suggesting that input from surface run-off and leaching was likely to be minimal. Furthermore, thiamethoxam use (area treated of arable crops) peaked in 2012 and has been followed by a decrease up to 2016 (Shardlow, 2017). For the biota samples, acetamiprid was infrequently detected in the Waveney, but consistently detected in the catchments of Alde, Deben, Gipping and Stour. However, this compound was often below the LOQ and upon quantification showed concentrations ranging from 0.2 to 0.7 ng g^−1^. Thiacloprid was frequently measured in the river Waveney and Deben with average concentrations of 3.3 ± 1.6 ng g^−1^ and 1.6 ± 1.7 ng g^−1^. With so little data available, meaningful comparisons of neonicotinoid concentrations with other pesticides in biota samples is difficult. Nonetheless, concentrations measured here were in the range to that of a previous investigation with thiacloprid ranging from LOQ – 21 ng g^−1^. Out of 10 pesticides that no longer have approval in the EU (Commission, E, 2009), a total of seven were detected in biota samples here (ametryn, dimethametryn, fenuron, propazine, aclonifen and oxycarboxine), including three that were quantifiable (ametryn, dimethametryn, fenuron). The most widespread occurrence corresponded to fenuron (0.7–16.1 ng g^−1^), oxycarboxine (qualitative) and ametryn (LOQ – 1.9 ng g^−1^). The compound oxycarboxine was detected with 100% frequency (Table S5) and fenuron with 86% frequency in biota samples. Detection of banned pesticides has recently been reported with atrazine (banned since 2003) quantified in 63% of samples (Bernard et al., 2019). However, there is little occurrence data available for the banned pesticides detected here, but several banned pesticides including fenuron, atrazine and simazine have been found to occur in UK groundwaters (Forum, 2015). The detection of these compounds in the environment might be explained by persistence and subsequent release of these compounds in sediments and/or soil (Bernard et al., 2019).

### 3.3 Estimating the toxic or effect pressure of contaminants in the aquatic environment

**Fig. 5:**
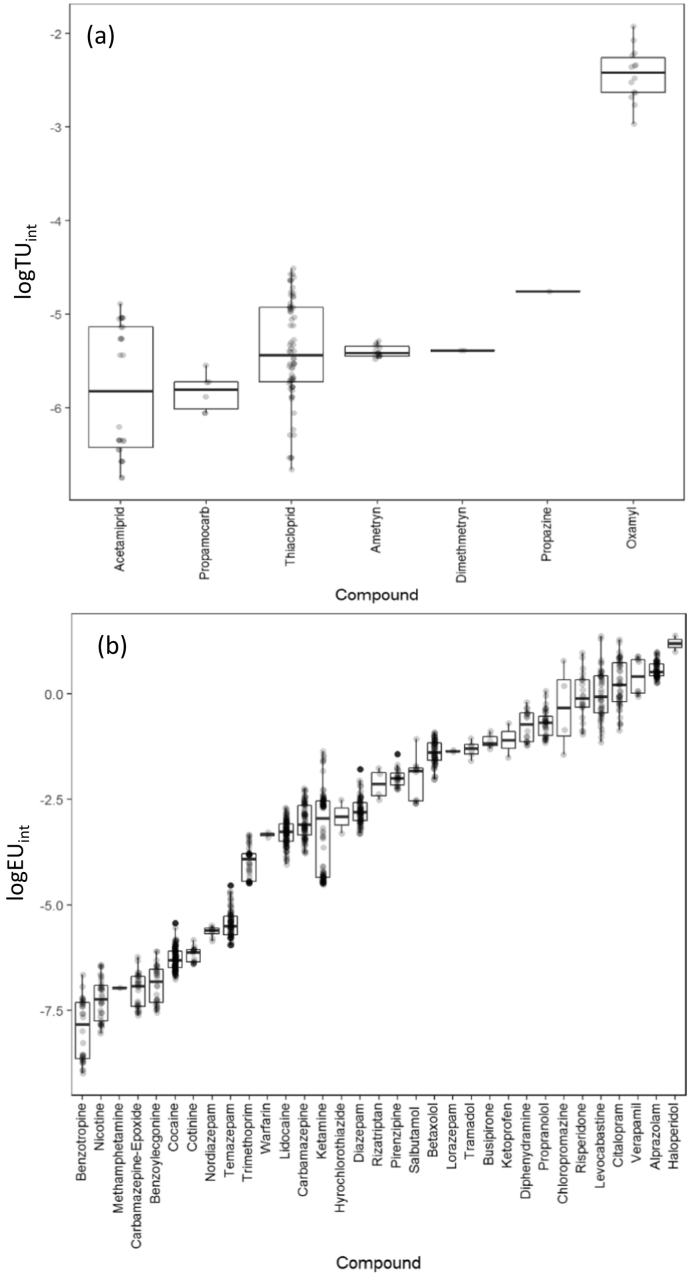
(a) Toxic pressure analysis of measured pesticides quantified by internal toxic units (logTU) (b) effect pressure analysis of measured pharmaceuticals and illicit drugs quantified by internal effect units (logEU_int_).

It has been suggested that internalised concentrations of contaminants are more appropriate for the assessment of potential risk in the environment than effect thresholds based on external exposure (i.e. in the water) (Miller et al., 2018). From the data here, we estimated the internal toxic pressure (pesticides) or ‘effect pressure’ (pharmaceuticals/drugs of abuse) (Munz et al., 2018) using predicted bioconcentration data (Miller et al., 2019; Agency, U.S.E.P., 2019) and the available effect data (EC_50_ or CEC) (Fick et al., 2010; Hertfordshire, 2019). This approach is analogous to risk quotients (RQ) estimated from predicted environmental concentrations and predicted no effect concentration (PEC/PNEC). The logTU_int_ for the pesticides determined ranged from approximately −7 to −2 (Fig. 5a), where previous studies have indicated that a logTU threshold based on water concentrations for pesticides of −3 and higher can elicit adverse effects (Beketov et al., 2009; Schäfer et al., 2007; Liess and Ohe, 2005), Only one compound (oxamyl) was above the threshold of logTU ≥ −3. This compound is still approved for use in the EU and may indicate the potential for risk at the concentrations measured in the biota samples. The EC_50_ was based on *D. magna* acute toxicity studies which have been shown to be the most sensitive across all aquatic organisms that were tested. However, the risk based on available evidence was concluded to be low (Authority, E.F.S, 2005). The neonicotinoids acetamiprid and thiacloprid showed low logTU_int_ values of less than −4.6. In comparison, Munz et al. (Munz et al., 2018) estimated thiacloprid to have a higher logTU_int_ in *G. pulex* than reported here and exceeded the threshold for several of the measured samples. The disparity between the estimation of toxic pressure is that concentrations of thiacloprid determined here in *G. pulex*, were relatively lower. In addition, the EC_50_ value used in this study was ~10-fold larger than in the previous study. For this approach EC_50_ data is often not well distributed and can vary depending on the end point, experimental conditions and species used. For these reasons, it may be more appropriate to include a range of the EC_50_ data available or review the quality of the available literature data to give more reliable estimation of toxic pressure (Küster et al., 2009).

The logTU threshold value is not likely to be directly applicable to pharmaceuticals, which are likely to be less toxic than pesticides by nature of their design. Thus, for this work we use the term ‘effect units’ (EU_int_) for pharmaceuticals, as thresholds that might be associated to toxicity are unknown. Instead, CEC data are used instead of EC50, but in themselves are not a toxicity endpoint. Substantial further work would be needed to determine possible thresholds associated with TU for different contaminant classes and for internalised concentrations, as opposed to surface water concentrations. Larger effect pressures were mainly associated with pharmaceuticals such as haloperidol that showed the highest EU_int_ (Fig. 5b). The reason haloperidol has high EU_int_ values is due to the low CEC of 6.5 ng L^−1^ based on human therapeutic plasma concentrations of 1 ng mL^−1^. Additional antipsychotic drugs including chloropromazine (CEC = 36 ng L^−1^) and risperidone (CEC = 129 ng L^−1^) were also estimated to have a high toxic pressure. Other neuroactive pharmaceuticals including antidepressants and anxiolytics such as alprazolam, lorazepam, citalopram and busipirone also showed higher EU_int_ which may indicate that these types of contaminants have a greater risk in the environment which has been previously suggested from surface water risk assessments (Sanderson et al., 2004). This may be particularly apparent when focussing on sub-lethal endpoints such as altered behaviour phenotypes (Fong and Ford, 2014). Despite its widespread occurrence, cocaine showed a low potential for an effect based on its CEC and BCF. The benefits of using CECs for pharmaceuticals is that the availability of data for human therapeutic values is greater than ecotoxicological data. In particular, EC_50_ data for ‘legacy’ pharmaceuticals is critically lacking. However, the use of CECs has some limitations in that a therapeutic effect does not necessarily correspond to an adverse effect and that the onset of pharmacological action may differ between humans and non-target organisms (Fick et al., 2010; Roos et al., 2012). Furthermore, molecular targets of pharmacological action are not always conserved between species and bioavailability may also differ between them (Cook et al., 2003; Roos et al., 2012).

## 4 Conclusion

Cocaine was the most widespread contaminant found in both surface water and biota samples, but no conclusions can be drawn about the potential for adverse effects of this compound without further work. Out of 67 compounds that could be quantitatively determined 56 were measured with the higher frequencies of detection for cocaine (100%), lidocaine (95%), alprazolam (88%), fenuron (86%) and ketamine (76%) in biota samples. In comparison for surface water samples, 50 compounds were measured including cocaine, carbamazepine, fenuron, ketamine and lidocaine, propranolol and tramadol that all had 100% detection frequency. The detection of several pesticides that no longer have approval in the EU warrants further investigation, as the sources for their input into the environment remain unclear. The total body burden of the contaminants determined in the biota samples ranged from 6.5 ng g^−1^ to 163.5 ng g^−1^ dw depending on the site. The total body burden is also an underestimate when accounting for the qualitative data, in addition to contaminants that were not targeted for in this study (including biotransformation products). Overall, whilst toxic pressure and effect pressure estimates were low in this study, the contribution of total body burden, the variability in effect data available (including lack of internal effect data) and thresholds for toxic/effect pressure are limitations to improving environmental risk assessment based on this approach. Nevertheless, the approach does support prioritisation of contaminants in the environment through the use of biomonitoring to reveal both the exposure, hazard and, ultimately, risk.

## Appendix A Supplementary data

Supplementary material Image 1

## References

[bb0005] Aboulfadl K. (2010). Time-dependent integrity during storage of natural surface water samples for the trace analysis of pharmaceutical products, feminizing hormones and pesticides.

[bb0010] Agency, U.S.E.P (2019). Estimation Programs Interface Suite™ for Microsoft® Windows, v 4.11.

[bb0015] Althakafy J.T. (2018). Determination of selected emerging contaminants in freshwater invertebrates using a universal extraction technique and liquid chromatography accurate mass spectrometry. J. Sep. Sci..

[bb0020] Álvarez-Muñoz D. (2015). Occurrence of pharmaceuticals and endocrine disrupting compounds in macroalgaes, bivalves, and fish from coastal areas in Europe. Environ. Res..

[bb0025] Authority, E.F.S (2005). Conclusion regarding the peer review of the pesticide risk assessment of the active substance oxamyl. EFSA J..

[bb0030] Baker D.R., Kasprzyk-Hordern B. (2011). Critical evaluation of methodology commonly used in sample collection, storage and preparation for the analysis of pharmaceuticals and illicit drugs in surface water and wastewater by solid phase extraction and liquid chromatography–mass spectrometry. J. Chromatogr. A.

[bb0035] Barat S.A., Abdel-Rahman M.S. (1996). Cocaine and lidocaine in combination are synergistic convulsants. Brain Res..

[bb0040] Barceló D. (1991). Occurrence, handling and chromatographic determination of pesticides in the aquatic environment. A review. Analyst.

[bb0045] Barron L., Tobin J., Paull B. (2008). Multi-residue determination of pharmaceuticals in sludge and sludge enriched soils using pressurized liquid extraction, solid phase extraction and liquid chromatography with tandem mass spectrometry. J. Environ. Monit..

[bb0050] Baz-Lomba J.A., Reid M.J., Thomas K.V. (2016). Target and suspect screening of psychoactive substances in sewage-based samples by UHPLC-QTOF. Anal. Chim. Acta.

[bb0055] Beketov M.A. (2009). SPEAR indicates pesticide effects in streams — comparative use of species- and family-level biomonitoring data. Environ. Pollut..

[bb0060] Benowitz N.L., Hukkanen J., Jacob P., Henningfield J.E., London E.D., Pogun S. (2009). Nicotine chemistry, metabolism, kinetics and biomarkers. Nicotine Psychopharmacology.

[bb0065] Bernard M. (2019). Combination of passive and grab sampling strategies improves the assessment of pesticide occurrence and contamination levels in a large-scale watershed. Sci. Total Environ..

[bb0070] Boillot C. (2015). In vivo exposure of marine mussels to carbamazepine and 10-hydroxy-10,11-dihydro-carbamazepine: bioconcentration and metabolization. Sci. Total Environ..

[bb0075] Bossus M.C. (2014). Behavioural and transcriptional changes in the amphipod Echinogammarus marinus exposed to two antidepressants, fluoxetine and sertraline. Aquat. Toxicol..

[bb0080] Brodin T. (2013). Dilute concentrations of a psychiatric drug Alter behavior of fish from natural populations. Science.

[bb0085] Bueno M.J.M. (2012). Occurrence and persistence of organic emerging contaminants and priority pollutants in five sewage treatment plants of Spain: two years pilot survey monitoring. Environ. Pollut..

[bb0090] Capaldo A. (2012). Presence of cocaine in the tissues of the European eel, Anguilla anguilla, exposed to environmental cocaine concentrations. Water Air Soil Pollut..

[bb0095] Carson R. (2002). Silent spring.

[bb0100] Carter L.J. (2019). Emerging Investigator Series: Towards a Framework for Establishing the Impacts of Pharmaceuticals in Wastewater Irrigation Systems on Agro-Ecosystems and Human Health.

[bb0105] Chang E.D. (2018). The use of molecular descriptors to model pharmaceutical uptake by a fish primary gill cell culture epithelium. Environmental Science & Technology.

[bb0110] Commission, E (2004). 2006/507/EC: council decision of 14 October 2004 concerning the conclusion, on behalf of the European Community, of the Stockholm Convention on Persistent Organic Pollutants. Off. J. Eur. Union.

[bb0115] Commission, E (2009). Regulation (EC) no 1107/2009 of the European Parliament and of the council of 21 October 2009 concerning the placing of plant protection products on the market and repealing council directives 79/117/EEC and 91/414/EEC. Off. J. Eur. Union.

[bb0120] Commission, E (2011). Commission implementing regulation (EU) no 540/2011 of 25 May 2011 implementing regulation (EC) no 1107/2009 of the European Parliament and of the council as regards the list of approved active substances text with EEA relevance. Off. J. Eur. Union.

[bb0125] Cook J.C., Ericson J.F., Williams R.T. (2003). A theoretical model for utilizing mammalian pharmacology and safety data to prioritize potential impacts of human pharmaceuticals to fish AU - Huggett, D. B. Human and Ecological Risk Assessment: An International Journal.

[bb0130] Dodder N.G. (2014). Occurrence of contaminants of emerging concern in mussels (Mytilus spp.) along the California coast and the influence of land use, storm water discharge, and treated wastewater effluent. Mar. Pollut. Bull..

[bb0135] Evans S., Bagnall J., Kasprzyk-Hordern B. (2017). Enantiomeric profiling of a chemically diverse mixture of chiral pharmaceuticals in urban water. Environ. Pollut..

[bb0140] Fick J. (2010). Predicted critical environmental concentrations for 500 pharmaceuticals. Regul. Toxicol. Pharmacol..

[bb0145] Fong P.P., Ford A.T. (2014). The biological effects of antidepressants on the molluscs and crustaceans: a review. Aquat. Toxicol..

[bb0150] Forum P. (2015). Pesticides in the UK: The 2015 Report on the Impacts and Sustainable Use of Pesticides.

[bb0155] González-Alonso S. (2017). Occurrence of pharmaceutical, recreational and psychotropic drug residues in surface water on the northern Antarctic peninsula region. Environ. Pollut..

[bb0160] Grabicova K. (2014). Tissue-specific bioconcentration of antidepressants in fish exposed to effluent from a municipal sewage treatment plant. Sci. Total Environ..

[bb0165] Hertfordshire U.o (2019). pesticide properties database. https://sitem.herts.ac.uk/aeru/ppdb/en/index.htm.

[bb0170] Hou X. (2003). Bioconcentration and elimination of sulfamethazine and its main metabolite in sturgeon (*Acipenser schrenkii*). J. Agric. Food Chem..

[bb0175] Huber A., Bach M., Frede H.G. (2000). Pollution of surface waters with pesticides in Germany: modeling non-point source inputs. Agric. Ecosyst. Environ..

[bb0180] Huerta-Fontela M. (2008). Occurrence of psychoactive stimulatory drugs in wastewaters in north-eastern Spain. Sci. Total Environ..

[bb0185] Huggett D.B. (2002). Toxicity of select Beta adrenergic receptor-blocking pharmaceuticals (B-blockers) on aquatic organisms. Arch. Environ. Contam. Toxicol..

[bb0190] Inostroza P.A. (2016). Body burden of pesticides and wastewater-derived pollutants on freshwater invertebrates: method development and application in the Danube River. Environ. Pollut..

[bb0195] Jabeen F. (2015). Examining pyrethroids, carbamates and neonicotenoids in fish, water and sediments from the Indus River for potential health risks. Environ. Monit. Assess..

[bb0200] Junqué E. (2018). Drivers of the accumulation of mercury and organochlorine pollutants in Mediterranean lean fish and dietary significance. Sci. Total Environ..

[bb0205] Kasprzyk-Hordern B., Dinsdale R.M., Guwy A.J. (2008). The occurrence of pharmaceuticals, personal care products, endocrine disruptors and illicit drugs in surface water in South Wales, UK. Water Res..

[bb0210] Küster A. (2009). Regulatory demands on data quality for the environmental risk assessment of pharmaceuticals. Regul. Toxicol. Pharmacol..

[bb0215] Lajeunesse A. (2011). Distribution of antidepressants and their metabolites in brook trout exposed to municipal wastewaters before and after ozone treatment — evidence of biological effects. Chemosphere.

[bb0220] Liess M., Ohe P.C.V.D. (2005). Analyzing effects of pesticides on invertebrate communities in streams. Environ. Toxicol. Chem..

[bb0225] Masiá A. (2013). Screening of currently used pesticides in water, sediments and biota of the Guadalquivir River Basin (Spain). J. Hazard. Mater..

[bb0230] Meredith-Williams M. (2012). Uptake and depuration of pharmaceuticals in aquatic invertebrates. Environ. Pollut..

[bb0235] Miller T.H. (2015). Pharmaceuticals in the freshwater invertebrate, Gammarus pulex, determined using pulverised liquid extraction, solid phase extraction and liquid chromatography–tandem mass spectrometry. Sci. Total Environ..

[bb0240] Miller T.H. (2017). Uptake, biotransformation and elimination of selected pharmaceuticals in a freshwater invertebrate measured using liquid chromatography tandem mass spectrometry. Chemosphere.

[bb0245] Miller T.H. (2018). A review of the pharmaceutical exposome in aquatic fauna. Environ. Pollut..

[bb0250] Miller T.H. (2019). Prediction of bioconcentration factors in fish and invertebrates using machine learning. Sci. Total Environ..

[bb0255] Mills G.A. (2014). Measurement of environmental pollutants using passive sampling devices—an updated commentary on the current state of the art. Environmental Science: Processes & Impacts.

[bb0260] Morrissey C.A. (2015). Neonicotinoid contamination of global surface waters and associated risk to aquatic invertebrates: a review. Environ. Int..

[bb0265] Munro K. (2019). Evaluation of combined sewer overflow impacts on short-term pharmaceutical and illicit drug occurrence in a heavily urbanised tidal river catchment (London, UK). Sci. Total Environ..

[bb0270] Munz N.A. (2018). Internal concentrations in Gammarids reveal increased risk of organic micropollutants in wastewater-impacted streams. Environmental Science & Technology.

[bb0275] van Nuijs A.L.N. (2011). Sewage epidemiology — a real-time approach to estimate the consumption of illicit drugs in Brussels, Belgium. Environ. Int..

[bb0280] Office, M (2018). Summer 2018. https://www.metoffice.gov.uk/climate/uk/summaries/2018/summer.

[bb0285] Owen S.F. (2009). Uptake of propranolol, a cardiovascular pharmaceutical, from water into fish plasma and its effects on growth and organ biometry. Aquat. Toxicol..

[bb0290] Petrie B. (2016). Multi-residue analysis of 90 emerging contaminants in liquid and solid environmental matrices by ultra-high-performance liquid chromatography tandem mass spectrometry. J. Chromatogr. A.

[bb0295] Potter J.M., Donnelly A. (1998). Carbamazepine-10, 11-epoxide in therapeutic drug monitoring. Ther. Drug Monit..

[bb0300] Richmond E.K. (2018). A diverse suite of pharmaceuticals contaminates stream and riparian food webs. Nat. Commun..

[bb0305] Roos V. (2012). Prioritising pharmaceuticals for environmental risk assessment: towards adequate and feasible first-tier selection. Sci. Total Environ..

[bb0310] Ryu Y. (2016). Comparative measurement and quantitative risk assessment of alcohol consumption through wastewater-based epidemiology: an international study in 20 cities. Sci. Total Environ..

[bb0315] Sanderson H. (2004). Ranking and prioritization of environmental risks of pharmaceuticals in surface waters. Regul. Toxicol. Pharmacol..

[bb0320] Schäfer R.B. (2007). Effects of pesticides on community structure and ecosystem functions in agricultural streams of three biogeographical regions in Europe. Sci. Total Environ..

[bb0325] Schultz M.M. (2010). Antidepressant pharmaceuticals in two U.S. effluent-impacted streams: occurrence and fate in water and sediment, and selective uptake in fish neural tissue. Environmental Science & Technology.

[bb0330] Sehonova P. (2017). Toxicity of naproxen sodium and its mixture with tramadol hydrochloride on fish early life stages. Chemosphere.

[bb0335] Shahid N. (2018). Pesticide body burden of the crustacean Gammarus pulex as a measure of toxic pressure in agricultural streams. Environmental Science & Technology.

[bb0340] Shardlow M. (2017). Neonicotinoid Insecticides in British Freshwaters.

[bb0345] Varol M., Sünbül M.R. (2017). Organochlorine pesticide, antibiotic and heavy metal residues in mussel, crayfish and fish species from a reservoir on the Euphrates River, Turkey. Environ. Pollut..

[bb0350] Yadav M.K. (2017). Occurrence of illicit drugs in water and wastewater and their removal during wastewater treatment. Water Res..

[bb0355] de Zwart D. (2001). Observed Regularities in Species Sensitivity Distributions for Aquatic Species, in Species Sensitivity Distributions in Ecotoxicology.

