# Supplementary material for: Biomonitoring of pesticides, pharmaceuticals and illicit drugs in a freshwater invertebrate to estimate toxic or effect pressure
Source: Environ Int. 2019 Aug;129:595–606. doi: 10.1016/j.envint.2019.04.038 (PMC6554641; doi:10.1016/j.envint.2019.04.038)
Supplement: Supplementary file 1 — Supplementary material [file mmc1.docx]

*Supplementary Information*

**BIOMONITORING OF PESTICIDES, PHARMACEUTICALS AND ILLICIT DRUGS IN A FRESHWATER INVERTEBRATE TO ESTIMATE TOXIC OR EFFECT PRESSURE**

*Thomas H. Miller^a^*, Keng Tiong Ng^a^, Samuel T. Bury^b^, Sophie E. Bury^c^, Nicolas R. Bury^d,e†,^ Leon P. Barron^a†^*

*^a^Department of Analytical, Environmental & Forensic Sciences, School of Population Health & Environmental Sciences, Faculty of Life Sciences and Medicine, King’s College London, 150 Stamford Street, London, SE1 9NH, UK.*

*^b^St Olaves Grammer School, Goddington Lane, Orpington, BR6 9SH, UK*

*^c^Department of Pyschology, Royal Holloway, University of London, Egham, Surrey TW20 0EX, UK*

*^d^School of Science, Technology and Engineering, University of Suffolk, James Hehir Building, University Avenue, Ipswich, Suffolk, IP3 0FS, UK.*

*^e^Suffolk Sustainability, University of Suffolk, Waterfront Building, Neptune Quay, Ipswich, IP4 1QJUK.*

*Corresponding author email: [thomas.miller@kcl.ac.uk](mailto:thomas.miller@kcl.ac.uk);

^†^Principal Investigators

Tel: +44 20 7848 4978

Table of Contents

**S 1.0** Analytical Standards……………………………………………………………………….S3

**S 2.0** Instrumental Conditions……………………………………………………………...……S4

**S 3.0** Method development……………………………………………………………………....S8

**S 4.0** Site Information ……………………………………………………………………..…...S11

**S 5.0** Bioconcentration Factor Prediction…………………………………………...………….S12

**S 6.0** Qualitative Detections……………………………………………………………………S14

**S 7.0** Example EICs………………………………………………………………………….…S18

*List of Tables*

**Table S1.** Overview of mass spectrometer conditions.

**Table S2.**

**Table S3.** Comparison of recovery and precision for selected analytes spiked at 50 ng g-1 using either ultrasonic extraction (USE) or pressurised liquid extraction (PLE) followed by tandem SPE.

**Table S4.** Overview of site information where animal and surface water samples were collected from.

**Table S5.** Prediction of bioconcentration factors (BCFs) using the US EPA EPI suite and a previously developed artificial neural network.

**Table S6.** Qualitative detection of compounds in animal samples collected across Suffolk that did not pass method validation criteria.

**Table S7.** Qualitative detection of compounds in surface water samples collected across Suffolk that did not pass method validation criteria

*List of Figures*

**Figure S1.** Extracted ion chromatogram examples in animal samples showing (a) fenuron standard at 10 ng g^-1^ versus sample (b) oxycarboxine standard versus sample (qualitative) (c) ametryn standard at 1 ng g^-1^ versus sample (d) cycluron standard at 1 ng g^-1^ versus sample (e) dimethametryn standard at 1 ng g^-1^ versus sample (f) mobile phase blank to assess any carry over from cocaine (absent). All standards are in matrix, dotted line represents the sample peak and solid line represents the standard peak.

**S1. Analytical Standards**

Reference standards for metformin-HCl, amphetamine, salicylic acid, dimetridazole, methamphetamine, nicotine, methcathinone, fenuron, cyromazine, cotinine, mephedrone, memantine-HCl, 4-fluoromethcathinone, propamocarb, antipyrine, 4-methylethcathinone, methedrone, 3,4-Methylenedioxymethamphetamine (MDMA), cymoxanil, cycluron, tacrine, ronidazole, simazine, levamisole-HCl, ibuprofen, methylone, ethirimol, clofibric acid, atrazine, oxamyl, acetamiprid, prometon, ametryn, propazine, diuron, methylphenidate-HCl, lidocaine, carboxine, carbamazepine, ketamine, mefenamic acid, bupropion-HCl, salbutamol, gemfibrozil, clothiandin, sulfapyridine, sulfadiazine, carbamazepine-10,11-epoxide, thiacloprid, sulfamethoxazole, ketoprofen, sulfathiazole, imidacloprid, dimethametryn, diphenhydramine-HCl, propranolol, tramadol-HCl, nortriptyline, sulfamerazine, aclonifen, sulfisoxazole, oxycarboxine, metoprolol, orphenadrine, mephosfolan, rizatriptan, nitenpyram, nordiazepam, flutamide, clotrimazole, venlafaxine-HCl, amitriptyline, sulfamethazine, sulfamonomethoxine, diazepam, morphine, oxazepam, ethofumesate, benzoylecgonine (BZE), thiamethoxam, diclofenac, meclofenamic acid, hydrochlorothiazide, norfluoxetine, carazolol, norethisterone, temazepam, cocaine, sertraline, warfarin, betaxolol, benzotropine, alprazolam, nadolol, fluoxetine, ketotifen, sulfadimethoxine, levonorgestrel, oxycodone-HCl, timolol, cocaethylene, chloropromazine-HCl, norfloxacin, lorazepam, clopidogrel-HSO4^-^, pyripoxyfen, flutolanil, citalopram-HBr, bisoprolol, cycloxidim, clozapine, paroxetine-HCl, nifedipine, medroxyprogesterone, prodiamine, lomefloxacin-HCl, pirenzepine-diHCL, piperophos, indomethacin, bezafibrate, fenofibrate, ofloxacine, enrofloxacin, fenoxaprop-p-ethyl, picoxystrobin, isradipine, fleroxacin, famoxadone, enalapril, 6a-methylprednisolone, haloperidol, cyphenothrin, celecoxib, sarafloxacin-HCl, busipirone-HCl, pyraclostrobin, dimethomorph, meclizine-HCl, bensulide, thiazopyr, difloxacin-HCl, azoxystrobin, lincomycin-HCl, amlodipine, tamsulosin-HCl, risperidone, carfentrazone-ethyl, ziprasidone-HCl, miconazole, cilazapril, levocabastine-HCl, valsartan, verapamil-HCl, oxytetracycline-HCl, terfenadine, chlortetracycline, flufenoxuron, fluocinide, ketoconazole, atorvastatin, azelnidipine, amiodarone-HCl, spinosad A, spinosad D, clarithromycin, azithromycin, josamycin and spiramycin were all obtained from Sigma-Aldrich (Steinheim, Germany). Trimethoprim, caffeine, and naproxen were ordered from Fluka (Buchs, Switzerland). Stable isotope-labelled standards (SIL-IS) including amphetamine-d6, nicotine-d4, cotinine-d3, MDMA-d5, methylone-d3-HCl, ketamine-d4-HCl, notriptyline-d3-HCl, tramadol-^13^C1,d3-HCl, nordiazepam-d5, amitriptyline-d3-HCl, venlafaxine-d6-HCl, diazepam-d6, oxazepam-d5, BZE-d3, norfluoxetine-d6, temazepam-d5, cocaine-d3, sertraline-d3-HCl, fluoxetine-d6, lorazepam-d4, haloperidol-d4, risperidone-d4, clothiandin-d3 and thiamethoxam-d3 were ordered from Sigma-Aldrich. Celecoxib-d7, clarithromycin-d3, betaxolol-d7-HCl, cetirizine-d4, gemfibrozil-d6, metoprolol-d7-HCl, lidocaine-d10-HCl, nifedipine-d4, sulfamethazine-d4, verapamil-d3-HCl and trimethoprim-d3 were sourced from QMX (Essex, UK).

**S2. Instrumental Analysis**

Tandem MS analysis was staged into four segments across the chromatographic run with approximately 50 – 60 SRM transitions monitored per scan event. A dwell time of 5 ms was utilised with a minimum of 10 points per peak with a mass tolerance of ± 0.25 m/z units for all transitions monitored. Confirmation of the selected compounds was achieved using both retention time (within ± 15 s) and a single transition selected for analyte quantification. The target analytes in both surface waters and biota were quantified based on their peak areas relative to that of an isotopically-labelled internal standard (spiked at 100 ng g^-1^) calibration curve (in matrix) or, where unavailable, by external matrix-matched calibration. Animal sample calibration curves ranged from 1 - 50 ng g^-1^ (N = 4) whereas surface water calibration curves ranged from 10 - 100 ng L^-1^ (N = 3) and were pooled samples from all sites. All samples were run at random in the sequence and carry-over was assessed via injections of mobile phase A.

**Table S1:** Overview of mass spectrometer conditions.

| Electrospray ionisation conditions | | | |
| --- | --- | --- | --- |
| Desolvation temperature (^o^C) | 300 | | |
| Source temperature (^o^C) | 100 | | |
| Sheath Gas pressure (AU) | 50 | | |
| Auxilliary Gas Pressure (AU) | 10 | | |
|  | Positive ion mode | Negative ion mode | |
| Capillary (kV) | 3.50 | | 3.50 |
| Collision Pressure (AU) | 1.5 | | 1.5 |

For this study, method performance characteristics are presented for *G. pulex* only and followed ICH guidelines for analytical method validation [1]. Matrix-matched calibration curves were generated for biota to assess method linearity, which was determined using peak area from 1 to 500 ng g-1 (N ≥ 7 for each compound). Limits of detection (LODs) were determined as the lowest concentration of analyte which produced a signal-to-noise (S/N) ratio of 3:1. Limits of quantification (LOQs) were determined as that analyte concentration to give an S/N ratio of 10:1. Both LOD and LOQ were calculated using the slope of the compound response and the standard deviation of the response for blank samples (n=6). Method precision (intra-day) was determined by spiking samples at 100 ng g^-1^ (n=3), 50 ng g-1 (n=5) and 25 ng g^-1^ (n=3). Inter-day precision was determined by different operators across three days by spiking samples at 50 ng g^-1^ (day 1 = 5 replicates, day 2 & day 3 in triplicate). Method accuracy (intra-day) was determined by samples spiked at 25 ng g^-1^, 50 ng g^-1^ and 100 ng g^-1^ in triplicate. Recovery was determined by comparing spiked samples at 50 ng g^-1^ (n=5) and compared to sample extracts spiked post-extraction (n=5) at the expected final concentration. The measurement of ion suppression or enhancement in ESI–MS involved the comparison of sample extracts spiked post-extraction (50 ng g^-1^, n=5) to a standard in ultra-pure water (n=3).

**Table S2:** SRM transitions for target analytes and SIL-IS.

| **Compound** | **ESI Polarity** | **Precursor** | **Product 1** | **Product 2** | **CE1** | **CE2** | **S Lens** |
| --- | --- | --- | --- | --- | --- | --- | --- |
| 4-Fluoromethcationone | + | 182.0 | 149.2 | 164.1 | 20 | 15 | 72 |
| Acetimiprid | + | 223.0 | 125.8 | 98.7 | 20 | 35 | 63 |
| Alprazolam | + | 309.0 | 281.1 | 205.3 | 30 | 40 | 130 |
| Ametryn | + | 228.1 | 186.0 | 68.0 | 20 | 35 | 102 |
| Antipyrin | + | 189.2 | 131.3 | 104.0 | 25 | 25 | 99 |
| Benzotropine | + | 308.2 | 167.1 | 165.2 | 35 | 35 | 136 |
| Benzoylecgonine | + | 290.2 | 168.1 | 105.0 | 20 | 35 | 84 |
| Betaxolol | + | 308.2 | 116.2 | 158.9 | 20 | 25 | 128 |
| Bezafibrate | - | 359.9 | 274.2 | 154.0 | 15 | 35 | 93 |
| Bisoprolol | + | 326.1 | 116.1 | 73.9 | 20 | 30 | 123 |
| Busipirone | + | 386.4 | 122.1 | - | 35 | - | 134 |
| Carbamazepine | + | 237.3 | 194.2 | 179.2 | 20 | 35 | 96 |
| CBZ_epoxide | + | 252.9 | 180.1 | 210.2 | 35 | 15 | 74 |
| Chloropromazine | + | 318.9 | 85.9 | - | 20 | - | 109 |
| Citalopram | + | 325.0 | 262.3 | 109.1 | 20 | 35 | 128 |
| Cocaine | + | 304.1 | 182.2 | 81.9 | 20 | 35 | 108 |
| Cotinine | + | 177.2 | 80.1 | 98.2 | 25 | 20 | 93 |
| Cycluron | + | 199.1 | 71.9 | 68.8 | 25 | 30 | 75 |
| Diazepam | + | 284.9 | 193.1 | 154.2 | 35 | 25 | 144 |
| Dimethmetryn | + | 256.2 | 186.2 | 68.0 | 20 | 35 | 104 |
| Diphenydramine | + | 256.2 | 167.2 | 152.1 | 15 | 35 | 68 |
| Ethirimol | + | 210.2 | 140.2 | 98.3 | 25 | 30 | 128 |
| Fenuron | + | 165.0 | 72.1 | 77.2 | 20 | 35 | 65 |
| Flutamide | - | 275.0 | 201.9 | 186.0 | 35 | 40 | 109 |
| Haloperidol | + | 376.1 | 165.0 | 123.1 | 20 | 45 | 144 |
| Hyrochlorothiazide | - | 296.0 | 268.9 | 205.1 | 20 | 20 | 80 |
| Ketamine | + | 238.2 | 125.0 | 179.2 | 35 | 20 | 91 |
| Ketoprofen | + | 255.0 | 209.2 | 105.0 | 15 | 25 | 72 |
| Ketotifen | + | 310.2 | 96.1 | - | 25 | - | 108 |
| Levamisole | + | 205.3 | 178.2 | 123.2 | 20 | 35 | 91 |
| Levocabastine | + | 421.2 | 174.2 | 70.0 | 35 | 40 | 143 |
| Lidocaine | + | 235.2 | 86.1 | - | 20 | - | 95 |
| Lincomycin | + | 407.2 | 126.2 | 359.1 | 35 | 20 | 143 |
| Lorazepam | + | 320.9 | 275.2 | 303.1 | 25 | 15 | 109 |
| MDMA | + | 194.2 | 163.1 | 135.1 | 15 | 20 | 64 |
| Mephedrone | + | 178.0 | 160.3 | 145.1 | 15 | 20 | 72 |
| Mephosfolan | + | 270.0 | 139.9 | 168.1 | 20 | 25 | 88 |
| Methamphetamine | + | 150.0 | 91.2 | 119.1 | 25 | 10 | 58 |
| Methcathinone | + | 164.0 | 131.0 | 146.0 | 15 | 15 | 63 |
| Methedrone | + | 194.1 | 176.1 | 161.2 | 15 | 15 | 75 |
| Methylphenidate | + | 234.0 | 84.2 | 129.2 | 20 | 35 | 88 |
| Metoprolol | + | 268.1 | 191.2 | 116.0 | 20 | 20 | 105 |
| Nicotine | + | 163.3 | 130.2 | 132.2 | 20 | 15 | 71 |
| Nadolol | + | 310.0 | 254.1 | 236.1 | 20 | 20 | 83 |
| Nordiazepam | + | 271.1 | 140.0 | 165.0 | 35 | 35 | 135 |
| Oxamyl | + | 220.1 | 163.1 | 134.8 | 15 | 25 | 71 |
| Oxazepam | + | 287.0 | 241.2 | 269.1 | 20 | 15 | 106 |
| Pirenzipine | + | 352.2 | 113.0 | 251.8 | 20 | 25 | 119 |
| Prometon | + | 226.1 | 184.1 | 142.0 | 20 | 25 | 110 |
| Propamocarb | + | 189.1 | 101.8 | 74.0 | 20 | 30 | 74 |
| Propazine | + | 230.1 | 188.1 | 145.8 | 25 | 30 | 105 |
| Propranolol | + | 260.3 | 116.2 | 183.0 | 20 | 20 | 120 |
| Risperidone | + | 411.2 | 191.2 | - | 35 | - | 121 |
| Rizatriptan | + | 270.1 | 201.2 | 158.2 | 15 | 25 | 67 |
| Salbutamol | + | 240.2 | 148.2 | 222.3 | 20 | 10 | 76 |
| Sulfadimethoxine | + | 311.0 | 156.0 | 108.2 | 20 | 35 | 122 |
| Sulfamethazine | + | 279.0 | 186.1 | 156.1 | 20 | 20 | 84 |
| Sulfapyridine | + | 250.0 | 156.1 | 184.1 | 15 | 20 | 98 |
| Tacrine | + | 199.2 | 171.2 | 144.1 | 35 | 40 | 161 |
| Tamsulosin | + | 409.1 | 228.2 | 300.0 | 25 | 35 | 149 |
| Temazepam | + | 300.9 | 255.2 | 283.2 | 25 | 15 | 108 |
| Thiacloprid | + | 253.0 | 98.1 | 126.1 | 20 | 45 | 98 |
| Timolol | + | 317.1 | 261.2 | 244.2 | 15 | 25 | 112 |
| Tramadol | + | 264.0 | 58.1 | - | 15 | - | 65 |
| Trimethoprim | + | 291.2 | 261.1 | 230.3 | 25 | 25 | 147 |
| Verapamil | + | 455.3 | 303.2 | 165.1 | 25 | 35 | 191 |
| Warfarin | - | 307.2 | 160.9 | 250.2 | 20 | 25 | 82 |
| *SIL-IS* |  |  |  |  |  |  |  |
| Gemfibrozil d6 | - | 255.2 | 120.8 | - | 15 | - | 71 |
| Nifedipine d4 | - | 348.9 | 222.3 | 125.8 | 10 | 15 | 91 |
| Methylone d3 | + | 210.9 | 163.2 | 135.0 | 20 | 35 | 69 |
| Haloperidol d4 | + | 380.1 | 169.2 | 127.0 | 25 | 35 | 149 |
| Nortriptyline d3 | + | 267.0 | 233.2 | 91.1 | 15 | 25 | 109 |
| Lorazepam d4 | + | 324.9 | 307.2 | 279.0 | 15 | 25 | 126 |
| Lidocaine d10 | + | 245.0 | 96.2 | 63.9 | 20 | 35 | 88 |
| Oxazepam d5 | + | 291.9 | 274.0 | 246.0 | 15 | 25 | 53 |
| Amphetamine d6 | + | 141.9 | 125.1 | 93.0 | 10 | 20 | 51 |
| Cocaine d3 | + | 307.0 | 185.1 | 85.2 | 20 | 35 | 114 |
| MDMA d5 | + | 198.9 | 165.1 | 135.2 | 15 | 20 | 58 |
| Tramadol 13C1,d3 | + | 268.0 | 58.0 | 249.9 | 15 | 15 | 81 |
| Verapamil d3 | + | 458.4 | 165.0 | 306.2 | 35 | 25 | 152 |
| Clarithromycin d3 | + | 751.4 | 593.4 | 160.9 | 20 | 35 | 172 |
| Trimethoprim d3 | + | 294.3 | 230.0 | 264.1 | 25 | 30 | 114 |
| Sertraline d3 | + | 308.9 | 275.1 | 158.9 | 15 | 35 | 71 |
| Betaxolol d7 | + | 315.1 | 123.2 | 161.2 | 20 | 25 | 131 |
| Methylphenidate d9 | + | 243.0 | 93.1 | - | 25 | - | 81 |
| Ketamine d4 | + | 241.9 | 224.3 | 129.1 | 15 | 35 | 79 |
| Risperidone d4 | + | 415.1 | 195.1 | - | 35 | - | 170 |
| BZE d3 | + | 293.0 | 171.1 | 105.1 | 20 | 35 | 109 |
| Celecoxib d7 | + | 389.0 | 369.1 | 289.3 | 30 | 35 | 170 |
| Metroprolol d7 | + | 275.1 | 133.0 | 123.0 | 35 | 20 | 111 |
| Sulfamethazine d4 | + | 282.9 | 112.0 | 185.8 | 35 | 20 | 120 |
| Norfluoxetine d6 | + | 302.7 | 140.0 | - | 10 | - | 57 |
| Nordiazepam d5 | + | 275.9 | 140.0 | 213.2 | 40 | 40 | 147 |
| Nicotine d4 | + | 166.9 | 134.0 | 121.1 | 20 | 35 | 71 |
| Fluoxetine d6 | + | 316.0 | 316.2 | 154.0 | 10 | 10 | 73 |
| Cotinine d3 | + | 179.9 | 79.9 | 101.0 | 45 | 30 | 76 |
| Temazepam d5 | + | 306.0 | 260.2 | 177.1 | 20 | 50 | 105 |
| Diazepam d6 | + | 290.0 | 198.2 | 89.5 | 50 | 60 | 133 |
| Amitriptyline d3 | + | 281.3 | 233.2 | 191.1 | 20 | 25 | 96 |
| Venlafaxine d6 | + | 284.1 | 63.9 | 121.1 | 45 | 45 | 80 |

**S3. Method Development**

The analytical method presented herein was developed using several different workflows to assess the optimal method in terms of precision, recovery, and resource use (i.e. time and cost). The workflows that were investigated included two different extraction methods, namely ultrasonic extraction (USE) and pressurised liquid extraction (PLE). Comparison of recovery and precision for the two extraction methods showed that USE had a higher average recovery and better repeatability than PLE for the selected analytes (**Table S3**). The average recovery and precision (%RSD) for USE and PLE were 63 ± 16% and 52 ± 24%, respectively (*t-*test, p = <0.006). Additionally, the USE method was significantly faster enabling up to 30 samples to be simultaneously extracted within 35 min in contrast to PLE that took 18 min per sample. Solvent use was also reduced with the USE method and was also a cheaper method of extraction than compared to PLE. Therefore, USE was selected and different clean-up and preconcentration strategies were subsequently compared to further optimise the analytical workflow. For sample clean-up, two different SPE cartridges were coupled together to help remove matrix interferences. The USE extraction with tandem SPE (HLB and Alumina-N) achieved better recovery (p = 0.044) and precision (p = 0.007) than USE with Oasis HLB alone (72 ± 11% and 63 ± 16%, respectively). However, even with the use of tandem SPE, matrix interferences were still significant (**Table 1**). Only four compounds showed matrix enhancement which corresponded to ketoprofen (10%), salbutamol (7%) nicotine (59%) and its metabolite cotinine (30%). All the remaining compounds showed suppression effects ranging from 10 – 82% suppression, with an average of 53% (median: -53%).Overall, the selected analytical method enabled 60 samples to processed across the day using traditional approaches to extract and sample clean-up. Based on method performance in Table 1 this workflow is fit-for-purpose and enabled reliable determination of internal concentrations of emerging contaminants. However, there is greater opportunities to increase throughput such as adapting these methods to 96-well plate extraction formats that would markedly reduce the amount of biomass required and time needed for sample preparation to improve throughput. Furthermore, advances in analytical capabilities are now enabling direct injection liquid chromatography-mass spectrometry more routinely circumventing the need for significant sample preparation [2]. Whilst this may not yet be appropriate for complex solid biological matrices, it is being used much more for complex liquid media such as surface and wastewater.

**Table S3:** Comparison of recovery and precision for selected analytes spiked at 50 ng g^-1^ using either ultrasonic extraction (USE) or pressurised liquid extraction (PLE) followed by tandem SPE.

|  | USE | | PLE | |
| --- | --- | --- | --- | --- |
| Compound | Recovery (%) | RSD | Recovery (%) | RSD |
|  | (n=6) | (%) | (n=6) | (%) |
| Sulfisoxazole | 86 | 10 | 2 | 66 |
| Sulfadiazine | 70 | 12 | 7 | 129 |
| Hyrochlorothiazide | 129 | 11 | 84 | 9 |
| Cotinine | 18 | 12 | 14 | 18 |
| Sulfamerazine | 48 | 11 | 21 | 32 |
| Sulfamonomethoxine | 57 | 13 | 29 | 20 |
| Sulfamethoxazole | 97 | 9 | 21 | 32 |
| Rizatriptan | 26 | 7 | 35 | 28 |
| Morphine | 6 | 33 | 34 | 15 |
| Sulfapyridine | 36 | 12 | 65 | 49 |
| BZE | 59 | 5 | 32 | 11 |
| Nadolol | 49 | 11 | 48 | 9 |
| Pirenzipine | 49 | 14 | 56 | 8 |
| Methcathinone | 16 | 12 | 19 | 27 |
| Methamphetamine | 48 | 13 | 98 | 104 |
| MDMA | 85 | 10 | 141 | 50 |
| Antipyrin | 102 | 13 | 31 | 12 |
| Sulfamethazine | 95 | 12 | 71 | 12 |
| Methedrone | 54 | 7 | 49 | 6 |
| Tacrine | 131 | 13 | 70 | 20 |
| Sulfadimethoxine | 48 | 18 | 52 | 15 |
| Trimethoprim | 44 | 16 | 56 | 14 |
| Mephedrone | 52 | 23 | 51 | 11 |
| Oxycodone | 65 | 12 | 77 | 7 |
| Levamisole | 50 | 12 | 87 | 17 |
| Timolol | 73 | 13 | 75 | 11 |
| Metoprolol | 71 | 11 | 0 | 0 |
| 4-methylethcathinone | 69 | 21 | 74 | 8 |
| Tramadol | 134 | 13 | 92 | 11 |
| Ketoprofen | 79 | 25 | 77 | 16 |
| Methylphenidate | 120 | 15 | 75 | 12 |
| Lincomycin | 12 | 9 | 74 | 8 |
| Warfarin | 55 | 56 | 59 | 39 |
| CBZ_epoxide | 161 | 10 | 99 | 9 |
| Bezafibrate | 25 | 35 | 87 | 20 |
| Cocaine | 91 | 11 | 62 | 12 |
| Carazolol | 52 | 19 | 30 | 25 |
| Venlafaxine | 95 | 19 | 86 | 54 |
| Bisoprolol | 56 | 13 | 71 | 14 |
| Levocabastine | 58 | 47 | 62 | 28 |
| Propranolol | 52 | 17 | 38 | 28 |
| Carbamazepine | 78 | 14 | 73 | 13 |
| Diclofenac | 77 | 76 | 141 | 140 |
| Risperidone | 50 | 16 | 37 | 35 |
| Ketotifen | 49 | 12 | 34 | 22 |
| Ketamine | 67 | 16 | 79 | 7 |
| Betaxolol | 66 | 14 | 56 | 21 |
| Diphenydramine | 59 | 13 | 32 | 22 |
| Oxazepam | 63 | 12 | 52 | 7 |
| Escitalopram | 52 | 12 | 34 | 27 |
| Lidocaine | 74 | 13 | 85 | 9 |
| Lorazepam | 68 | 10 | 49 | 10 |
| Alprazolam | 59 | 16 | 60 | 10 |
| Haloperidol | 45 | 11 | 28 | 8 |
| Busipirone | 70 | 8 | 46 | 8 |
| Orphenadrine | 57 | 13 | 33 | 28 |
| Temazepam | 65 | 3 | 55 | 8 |
| Nordiazepam | 43 | 25 | 41 | 20 |
| Nortriptyline | 53 | 8 | 34 | 6 |
| Benzotropine | 50 | 14 | 30 | 13 |
| Clozapine | 53 | 11 | 33 | 22 |
| Amitriptyline | 39 | 43 | 24 | 44 |
| Diazepam | 56 | 15 | 44 | 12 |
| Verapamil | 49 | 12 | 24 | 11 |
| Roxithromycin | 55 | 10 | 31 | 24 |
| Ziprasidone | 32 | 12 | 10 | 59 |
| Flutamide | 54 | 13 | 36 | 9 |
| Levonorgestrel | 69 | 15 | 45 | 14 |
| Josamycin | 71 | 12 | 23 | 20 |

**S4. Site Information**

**Table S4:** Overview of site information where animal and surface water samples were collected from.

**
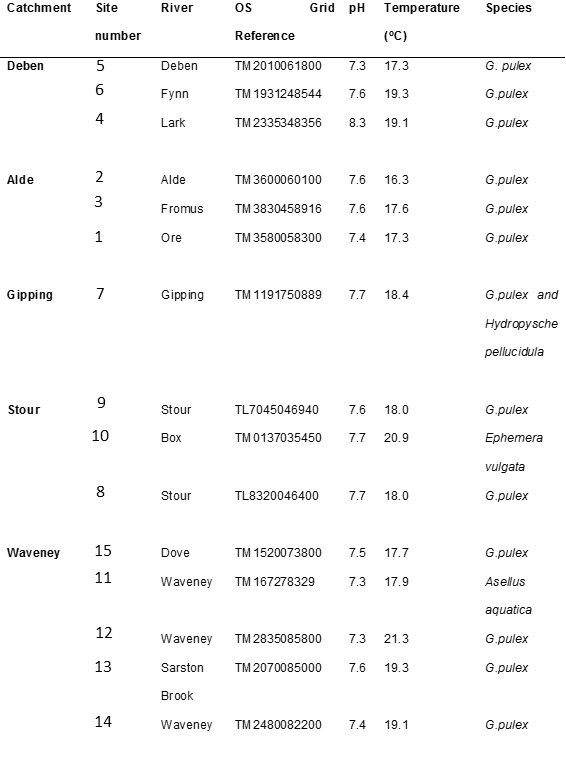
**

**S5. Bioconcentration Factor Prediction**

**Table S5:** Prediction of bioconcentration factors (BCFs) using the US EPA EPI suite and a previously developed artificial neural network.

| Compound | EPI Suite BCF | ANN BCF |
| --- | --- | --- |
| 4-fluoromethcathinone | 3 | 15 |
| Acetamiprid | 22 | 1 |
| Alprazolam | 12 | 12 |
| Ametryn | 13 | 0 |
| Antipyrin | 3 | 5 |
| Benzotropine | 311 | 6739 |
| Benzoylecgonine | 3 | 20 |
| Betaxolol | 18 | 21 |
| Bezafibrate | 3 | 20 |
| Bisoprolol | 4 | 22 |
| Busipirone | 25 | 20 |
| Carbamazepine | 19 | 18 |
| CBZ_epoxide | 3 | 15 |
| Chloropromazine | 1720 | 40 |
| Citalopram | 137 | 16 |
| Cocaine | 15 | 19 |
| Cotinine | 3 | 6 |
| Cycluron | 35 | 33 |
| Diazepam | 34 | 63 |
| Dimethmetryn | 53 | 0 |
| Diphenydramine | 67 | 14 |
| Ethirimol | 6 | 6 |
| Fenuron | 3 | 2 |
| Flutamide | 75 | 39 |
| Haloperidol | 83 | 34 |
| Hydrochlorothiazide | 3 | 19 |
| Imidacloprid | 3 | 9 |
| Ketamine | 13 | 841 |
| Ketoprofen | 3 | 20 |
| Ketotifen | 42 | 1243 |
| Levamisole | 8 | 2 |
| Levocabastine | 3 | 23 |
| Lidocaine | 19 | 9 |
| Lincomycin | 3 | 22 |
| Lorazepam | 18 | 20 |
| MDMA | 12 | 12 |
| Mephedrone | 5 | 19 |
| Mephosfolan | 2 | 23 |
| Methamphetamine | 11 | 0 |
| Methcathinone | 2 | 10 |
| Methedrone | 2 | 11 |
| Methylphenidate | 3 | 13 |
| Metoprolol | 5 | 20 |
| Nicotine | 3 | 0 |
| Nadolol | 3 | 22 |
| Nordiazepam | 40 | 47 |
| Oxamyl | 3 | 6 |
| Oxazepam | 14 | 19 |
| Pirenzipine | 6 | 11 |
| Prometon | 13 | 0 |
| Propamocarb | 3 | 6 |
| Propazine | 12 | 0 |
| Propranolol | 51 | 20 |
| Risperidone | 93 | 21 |
| Rizatriptan | 4 | 17 |
| Salbutamol | 3 | 19 |
| Sulfadimethoxine | 6 | 19 |
| Sulfamethazine | 3 | 16 |
| Sulfapyridine | 3 | 13 |
| Tacrine | 29 | 13 |
| Tamsulosin | 20 | 22 |
| Temazepam | 13 | 19 |
| Thiacloprid | 16 | 3 |
| Timolol | 4 | 17 |
| Tramadol | 25 | 18 |
| Trimethoprim | 3 | 14 |
| Verapamil | 147 | 22 |
| Warfarin | 28 | 21 |

**S6. Qualitative Detections**

**Table S6:** Qualitative detection of compounds in animal samples collected across Suffolk that did not pass method validation criteria.


**Table S7:** Qualitative detection of compounds in surface water samples collected across Suffolk that did not pass method validation criteria

**S7. Example EICs**


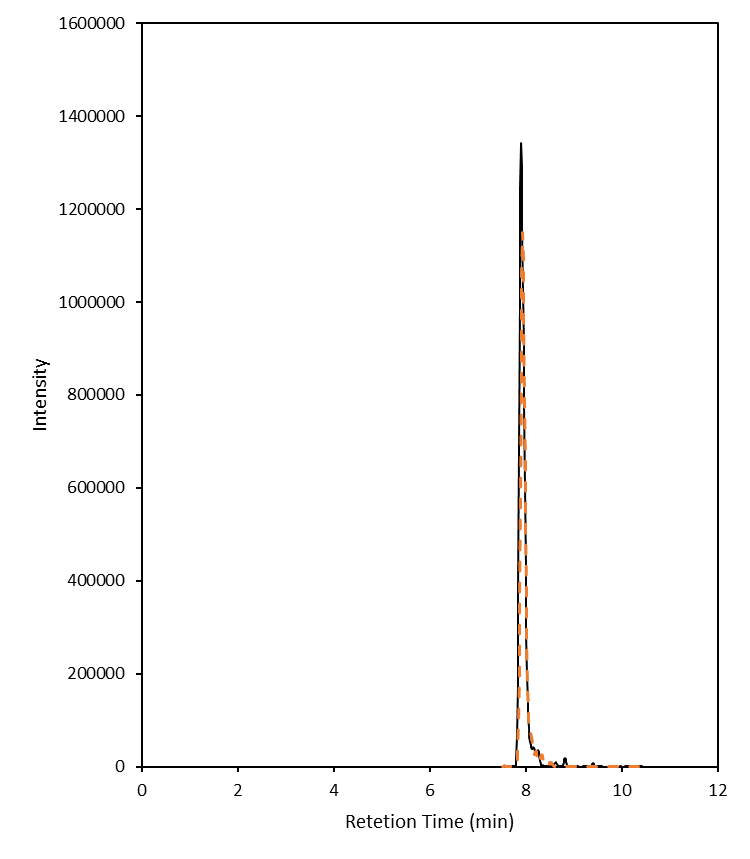

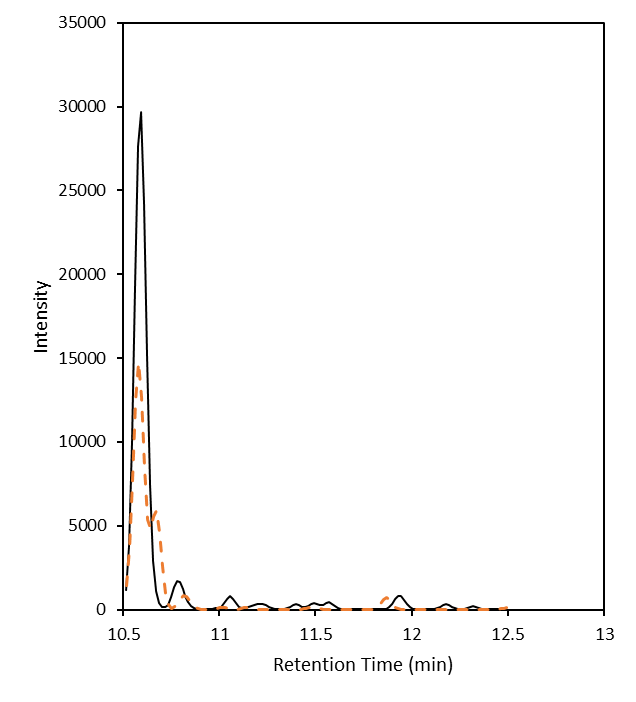

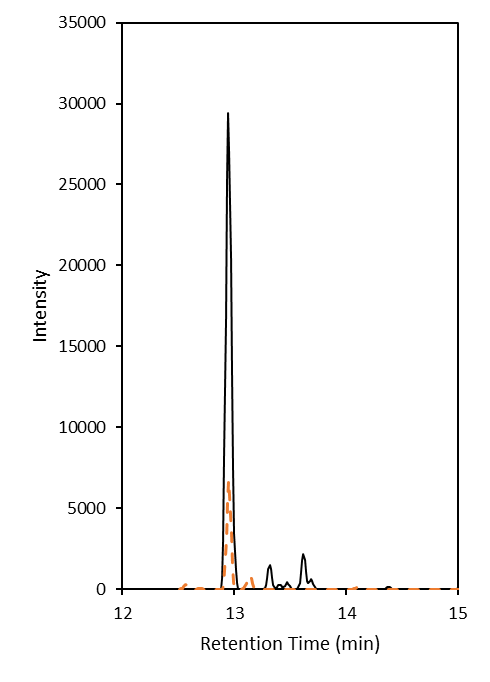

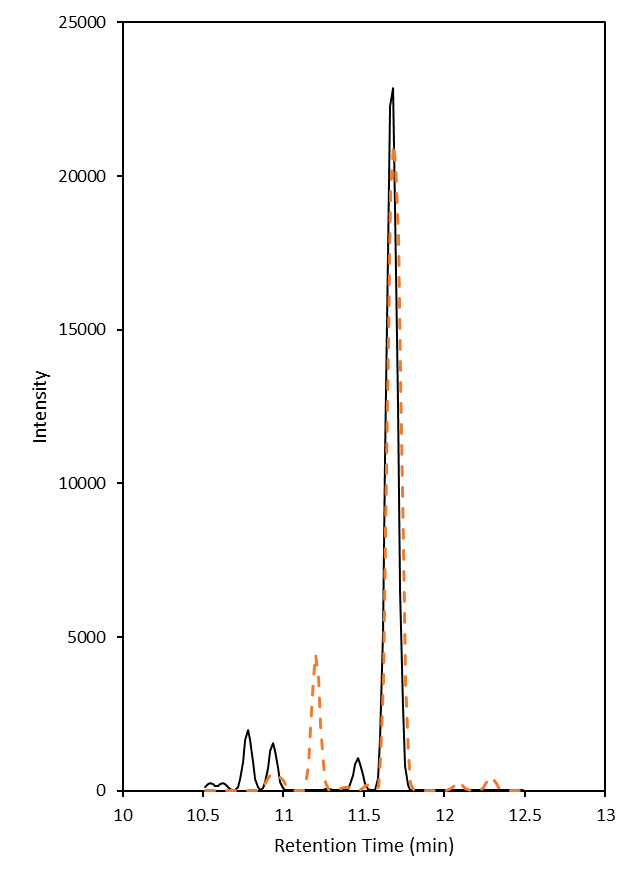

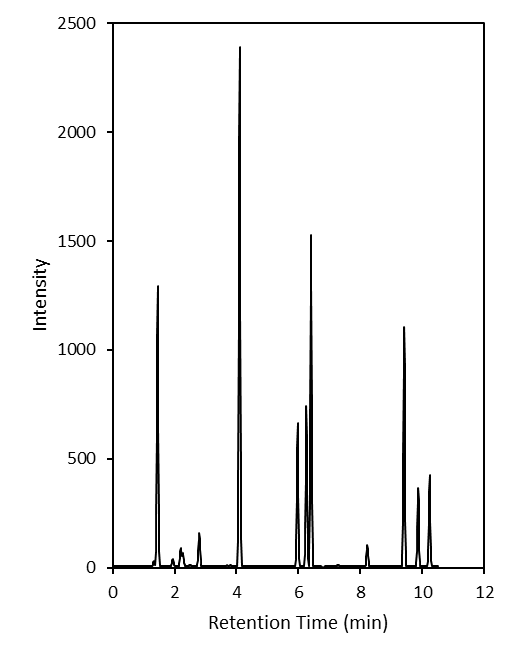

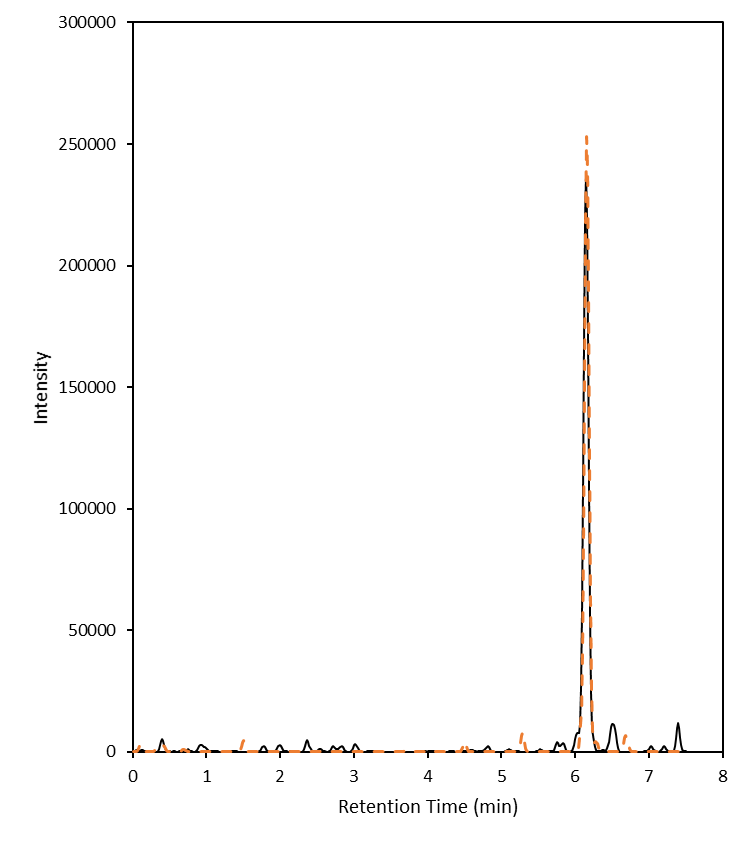


(b)

(c)

(d)

(e)

(f)

(a)

**Figure S2**: Extracted ion chromatogram examples in animal samples showing (a) fenuron standard at 10 ng g^-1^ versus sample (b) oxycarboxine standard versus sample (qualitative) (c) ametryn standard at 1 ng g^-1^ versus sample (d) cycluron standard at 1 ng g^-1^ versus sample (e) dimethametryn standard at 1 ng g^-1^ versus sample (f) mobile phase blank to assess any carry over from cocaine (absent). All standards are in matrix, dotted line represents the sample peak and solid line represents the standard peak.

**References**

1. Harmonization, I.C.o., *International Conference on Harmonization (ICH) of Technical Requirements for the Registration of Pharmaceuticals for Human use, Validation of Analytical Procedures, ICH Q2A* 1996: Geneva.

2. Oliveira, T.S., et al., *Characterization of Pharmaceuticals and Personal Care products in hospital effluent and waste water influent/effluent by direct-injection LC-MS-MS.* Science of The Total Environment, 2015. **518-519**: p. 459-478.
